# Synthesis of new Ag(i) and Cd(ii) hydrozanomide-based complexes with antibacterial and anticancer properties†

**DOI:** 10.1039/d5ra02001h

**Published:** 2025-06-10

**Authors:** Alina Climova, Ekaterina Pivovarova, Małgorzata Szczesio, Katarzyna Gobis, Agnieszka Korga-Plewko, Magdalena Iwan, Edyta Kordialik-Bogacka, Sylwia Ścieszka, Jaromir Marek, Agnieszka Czylkowska

**Affiliations:** a Institute of General and Ecological Chemistry, Faculty of Chemistry, Lodz University of Technology Zeromskiego 116 Lodz 90-924 Poland alina.climova@dokt.p.lodz.pl agnieszka.czylkowska@p.lodz.pl; b Department of Organic Chemistry, Faculty of Pharmacy, Medical University of Gdańsk 107 Gen. Hallera Ave. Gdańsk 80-416 Poland; c Independent Medical Biology Unit, Faculty of Pharmacy, Medical University of Lublin Jaczewskiego 8b Lublin 20-093 Poland; d Institute of Fermentation Technology and Microbiology, Faculty of Biotechnology and Food Sciences, Lodz University of Technology Wólczańska 171/173 Lodz 90-924 Poland; e Core Facility Biomolecular Interactions and Crystallography, CEITEC MU, Masaryk University Kamenice 5 Brno 62500 Czech Republic; f Department of Chemistry, Faculty of Science, Masaryk University Kamenice 5 Brno 62500 Czech Republic

## Abstract

A series of new Ag(i) and Cd(ii) hydrozanomide-based complexes have been prepared and studied. The synthesized compounds have undergone extensive spectroscopic and structural characterization, including F-AAS, FTIR, single crystal XRD and TGA analyses. Crystal structures of two representative complexes, 4 and 6, have revealed that the silver atom in complex 4 assumes a four-coordinate geometry with two ligands. Additionally, the stability of these complexes in DMSO has been determined using UV-vis spectroscopy. The biological activity of these metal complexes has been assessed, focusing on their antibacterial and anticancer properties against LN-229 and U87 cell lines. Among the tested compounds, silver-based complexes demonstrated significant antibacterial activity against a wide range of microorganisms. Moreover, cytotoxicity studies have shown that the metal complexes exhibit higher anticancer activity compared to their parent ligands. Notably, the Ag(i) complexes of L_3_, L_4_, and L_5_ have emerged as the most promising candidates for their selective toxicity against cancer cells, without harmful effects on normal human fibroblast cells. These findings highlight the potential of Ag(i) complexes as promising anticancer agents with selective toxicity and potent antibacterial properties.

## Abstract 2

Selective toxicity of the complex against cancer cells, without harmful effects on normal human fibroblast cells.

## Graphical abstract

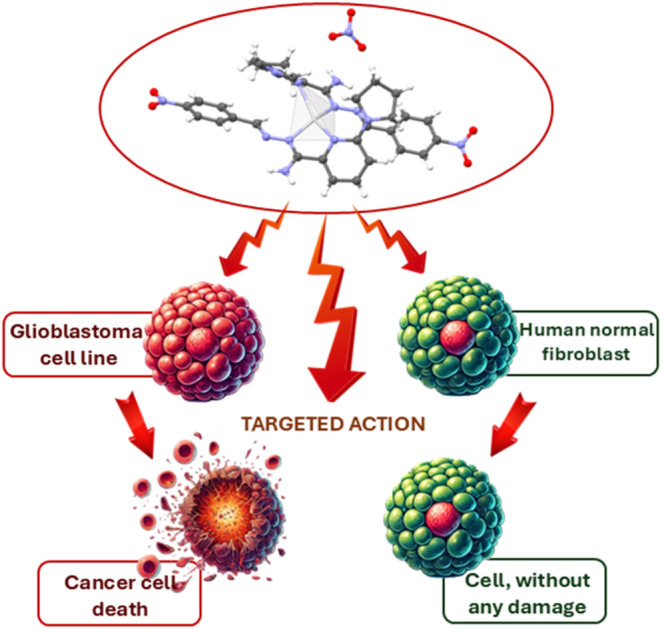

## Introduction

Coordination chemistry is a promising field that has several advantages in the development of new therapies for cancer treatment.^1^ Metal complexes, often derived from transition metals such as platinum, have shown promising anticancer properties. The most commonly used and effective ones are cisplatin, carboplatin, and oxaliplatin.^2^ The ability of metal ions to coordinate with biomolecules such as DNA underlies their effectiveness in inhibiting cell proliferation. The mechanism of action is attributed to their ability to penetrate the cell, bind to cellular DNA, forming cross-links that alter the DNA structure and disrupt its function.^3,4^ One of the major benefits of coordination compounds is their inherent tunability. This allows researchers to precisely adjust these compounds to specific biological targets, enhancing their selectivity and reducing side effects.^5^ Through precise compound design, researchers synthesize complexes that selectively interact with cancer cells, sparing healthy tissues and minimizing damage to normal cells.^6–9^ This targeted approach enhances therapeutic efficacy and minimizes the risk of adverse side effects. It also promotes safer and more efficient treatments, making coordination compounds an attractive option for the development of personalized medicine. The capacity to modify these compounds to optimize their pharmacokinetic and pharmacodynamic properties presents a significant opportunity to advance personalized treatment options. Although coordination compounds have promising therapeutic potential, many metal-based candidates, especially those already close to clinical application, have been discontinued due to unacceptable toxicity. For this reason, this area needs more extensive research and more expanded database.^10^

Ongoing research efforts around the world are aiming to reduce the side effects of chemotherapy through the development of novel complexes containing d^*n*^-electron metals as alternatives to platinum-based drugs.^11,12^ Silver has a more effective antimicrobial effect than penicillin and other antibiotics, and also causes a similar effect on antibiotic-resistant strains of bacteria.^13^ Budagumpi's team studied the antimicrobial properties of benzimidazolium salts and their bis-*N*-heterocyclic carbene silver(i) complexes. Some of the tested compounds showed excellent antibacterial activity against *E. coli*, with a MIC value of 4 μg mL^−1^, which is comparable to the standard antibiotic ampicillin. One of the compounds demonstrated a MIC value of 2–4 μg mL^−1^ against *S. aureus*, *E. coli*, and *P. aeruginosa*, which is better than the ampicillin value.^14,15^ Ag ions have a significant ability to inactivate influenza viruses, some entero- and adenoviruses, as well as inhibit the activity of the AIDS virus.^16^ Moreover, silver complexes exhibited more potent cytotoxicity with the least toxicity compared to cisplatin.^17^ Cd(ii) complexes have attracted attention due to the growing recognition of their role in biological processes and rich structural chemistry.^18–21^ Cadmium complexes showed anticancer activity against Mia Paca-2, BxPC-3 and Panc-1 pancreatic cancer cell lines with IC_50_ values up to 16.0 μM.^22^ In addition, it has been reported that complexes of this metal inhibit T-cell leukemia tumor growth in mice.^23^ Cadmium complexes have also attracted attention as potential antimicrobial agents.^24,25^ Cadmium can disrupt the functioning of bacterial cells by embedding into their membranes and disrupting their integrity, as well as inhibiting the activity of various bacterial enzymes, which disrupt their metabolic processes and lead to cell death.^26,27^

**Fig. 1:**
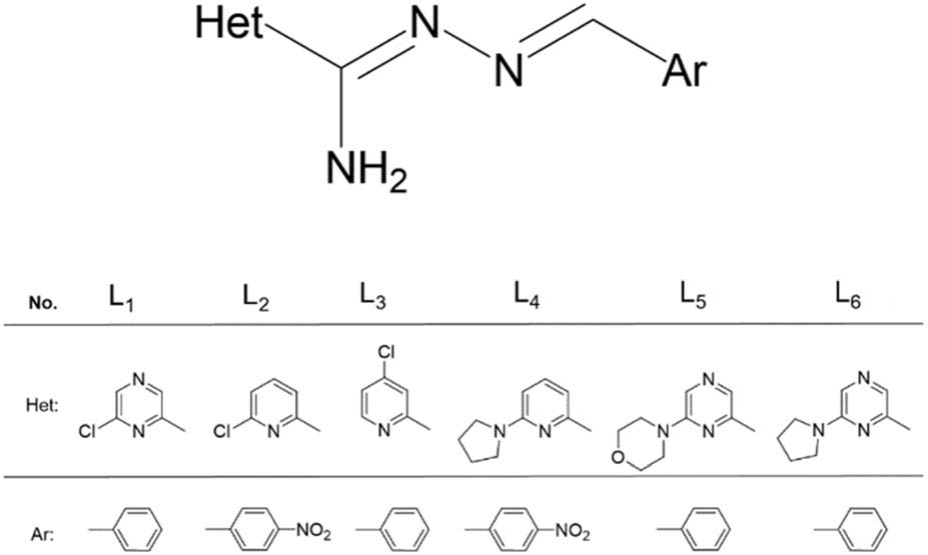
The structure of the ligands L_1_–L_6_.

Previously, we synthesized new organic pyridine and pyrazine based-ligands and a series of neutral Cu(ii) and Zn(ii) complexes based on them.^28,29^ The present work is a continuation of our previous studies on the biological activity of metal complexes, and herein, we report the synthesis, spectroscopic and structural characterization of them. We investigated new Ag(i) and Cd(ii) complexes based on the previously described ligands: L_1_ – *N*′-(benzylidene)-6-chloropyrazine-2-carbohydrazonamide, L_2_ – 6-chloro-*N*′-(4-nitrobenzylidene) picolinohydrazonamide, L_3_ – *N*′-(benzylidene)-4-chloropicolinohydrazonamide, L_4_ – (4-nitrobenzylidene)-6-(pyrrolidin-1-yl)picolinohydrazonamide, L_5_ – (benzylidene)-6-morpholinopyrazine-2-carbohydrazonamide and L_6_ – (benzylidene)-6-(pyrrolidin-1-yl)pyrazine-2-carbohydra-zonamide (Fig. 1).

All synthesized complexes were characterized by F-AAS, FTIR, UV-vis, and TGA analysis. Only for two coordination compounds (4 and 6) the single crystals were obtained. The crystal structures of complexes 4 and 6 were described, where Ag atom of complex 4 is four-coordinated by two ligand molecules. Additionally, antibacterial and anticancer activity of the obtained compounds were evaluated. Among all the tested compounds, silver complexes demonstrated the most promising results in almost all microorganisms used. Anticancer activity measurements revealed that synthesized metal complexes generally exhibited greater cytotoxic potential than their parent ligands, with varying sensitivity between different cell lines. The results were compared to the activity of the human skin fibroblast cell line BJ. The Ag complexes of L_3_, L_4_, and L_5_ were the most promising compounds, as they showed selective toxicity towards cancer cells while having no effect on normal cells.

## Result and discussion

### Synthesized complexes

#### Complex 1

AgL_1_NO_3_ × H_2_O (C_12_H_10_ClN_5_AgNO_3_H_2_O) 447.58 g mol^−1^. Light brown powder. Yield = 88.3%. M.p. = 188.6 °C. Anal. calculated (%): Ag, 24.1%; C, 32.2%; H, 2.7%; N, 18.78%. Found (%): Ag, 23.23%; C, 31.23%; H, 2.156%; N, 18.74%. FTIR spectra (KBr, cm^−1^): *ν*(NH) 3427, 3311; *ν*(C–H) 3052; *ν*(C=N) 1632; *δ*(NH) 1550; *β*(CH) 1366; *ν*(C=C) 1444; *ν*(N–N) 1169; *γ*(CH) 855.

#### Complex 2

AgL_2_NO_3_ (C_13_H_10_ClN_5_O_2_AgNO_3_) 473.58 g mol^−1^. Dark brown powder. Yield = 95.39%. M.p. = 186.3 °C. Anal. calculated (%): Ag, 22.78%; C, 32.97%; H, 2.13%; N, 17.75%. Found (%): Ag, 22.87%; C, 32.88%; H, 2.192%; N, 17.67%. FTIR spectra (KBr, cm^−1^): *ν*(NH) 3468, 3361; *ν*(C–H) 3102; *ν*(C=N) 1626; *δ*(NH) 1558; *β*(CH) 1382; *ν*(C=C) 1457; *ν*(N–N) 1160, 1130; *γ*(CH) 854.

#### Complex 3

AgL_3_NO_3_ (C_13_H_11_ClN_4_AgNO_3_) 428.58 g mol^−1^. Green powder. Yield = 64.26%. M.p. = 178.5 °C. Anal. calculated (%): Ag, 25.17%; C, 36.43%; H, 2.59%; N, 16.34%. Found (%): Ag, 25.55%; C, 35.36%; H, 2.57%; N, 16.03%. FTIR spectra (KBr, cm^−1^): *ν*(NH) 3393, 3288; *ν*(C–H) 3057; *ν*(C=N) 1622; *δ*(NH) 1560; *β*(CH) 1373; *ν*(C=C) 1445; *ν*(N–N) 1175, 1120; *γ*(CH) 881.

#### Complex 4

AgL_4_NO_3_ (C_34_H_36_N_12_O_6_AgNO_3_) 846.63 g mol^−1^. Light brown powder. Yield = 81.09%. M.p. = 182.3 °C. Anal. calculated (%): Ag, 12.28%; C, 46.48%; H, 4.13%; N, 20.72%. Found (%): Ag, 13.03%; C, 45.95%; H, 3.73%; N, 19.94%. FTIR spectra (KBr, cm^−1^): *ν*(NH) 3440, 3267; *ν*(C–H) 3079; *ν*(C=N) 1632; *δ*(NH) 1593; *β*(CH) 1360; *ν*(C=C) 1449; *ν*(N–N) 1165, 1006; *γ*(CH) 881.

#### Complex 5

AgL_5_NO_3_ × H_2_O (C_16_H_18_N_6_OAgNO_3_H_2_O) 498.24 g mol^−1^. Green powder. Yield = 72.27%. M.p. = 185.8 °C. Anal. calculated (%): Ag, 21.65%; C, 38.57%; H, 4.05%; N, 19.68%. Found (%): Ag, 22.46%; C, 37.7%; H, 3.87%; N, 19.27%. FTIR spectra (KBr, cm^−1^): *ν*(NH) 3403, 3288; *ν*(C–H) 3080; *ν*(C=N) 1629; *δ*(NH) 1573, 1525; *β*(CH) 1372; *ν*(C=C) 1447; *ν*(N–N) 1117, 1069; *γ*(CH) 885.

#### Complex 6

AgL_6_NO_3_ (C_16_H_18_N_6_AgNO_3_) 464.23 g mol^−1^. Green powder. Yield = 69.17%. M.p. = 198.6 °C. Anal. calculated (%): Ag, 23.24%; C, 41.4%; H, 3.91%; N, 21.12%. Found (%): Ag, 23.17%; C, 40.82%; H, 3.68%; N, 20.75%. FTIR spectra (KBr, cm^−1^): *ν*(NH) 3349, 3279; *ν*(C–H) 3063; *ν*(C=N) 1629; *δ*(NH) 1527, 1565; *β*(CH) 1377; *ν*(C=C) 1490, 1449; *ν*(N–N) 1147, 1024; *γ*(CH) 862.

#### Complex 7

Cd (L_2_)_2_Cl_2_ ((C_13_H_10_N_5_ClO_2_)_2_CdCl_2_) 790.728 g mol^−1^. Yellow powder. Yield = 55.83%. M.p. = 188.6 °C. Anal. calculated (%):Cd, 14.22%; C, 39.49%; H, 2.55%; N, 17.71%. Found (%): Cd, 14.14%; C, 40.06%; H, 2.67%; N, 18.54%. FTIR spectra (KBr, cm^−1^): *ν*(NH) 3480, 3374; *ν*(C–H) 3107; *ν*(C=N) 1624; *δ*(NH) 1557; *β*(CH) 1381; *ν*(C=C) 1456; *ν*(N–N) 1160, 1105; *γ*(CH) 854.

#### Complex 8

CdL_3_Cl_2_ (C_13_H_11_N_4_ClCdCl_2_) 442.023 g mol^−1^. Light yellow powder. Yield = 75.16%. M.p. = 250 °C. Anal. calculated (%): Cd, 25.43%; C, 35.32%; H, 2.51%; N, 12.68%. Found (%): Cd, 25.02%; C, 34.96%; H, 2.48%; N, 12.6%. FTIR spectra (KBr, cm^−1^): *ν*(NH) 3393, 3300; *ν*(C–H) 3095; *ν*(C=N) 1632; *δ*(NH) 1538; *β*(CH) 1376; *ν*(C=C) 1447; *ν*(N–N) 1186, 1123; *γ*(CH) 870.

#### Complex 9

CdL_4_Cl_2_ (C_17_H_18_N_6_O_2_CdCl_2_) 521.68 g mol^−1^. Orange powder. Yield = 71.12%. M.p. = 223.5 °C. Anal. calculated (%): Cd, 21.54%; C, 39.14%; H, 3.48%; N, 16.10%. Found (%): Cd, 21.03%; C, 40.1%; H, 3.79%; N, 15.71%. FTIR spectra (KBr, cm^−1^): *ν*(NH) 3410, 3306; *ν*(C–H) 3078; *ν*(C=N) 1630; *δ*(NH) 1558; *β*(CH) 1404; *ν*(C=C) 1457; *ν*(N–N) 1175, 1149; *γ*(CH) 857.

### FTIR

FTIR spectra of free ligands and Ag(i) and Cd(ii) coordination compounds are shown in Fig. S1.† The spectra of pure ligands have been described previously.^28,29^ The main frequencies and distribution are summarised in Table S1.†

The stretching vibration mode of the –NH– group is observed in the range of 3470–3300 cm^−1^, composed of two peaks. After complexation, these vibration modes are shifted and decreased in intensity. For complex 5, there is an overlap of the *ν*(NH) vibrations due to the presence of water molecules. In the range of 3100–3000 cm^−1^, stretching vibrations of CH groups are observed for all complexes.

The sharp CN bands observed in the range of 1632–1614 cm^−1^ (ref. 30–33) for the ligands shifted after complexation and did not change their intensity. A more significant shift was observed for complexes 8 and 9, which is proposed to be due to complexation *via* nitrogen from the pyridine/pyrazine ring. Complexes 4, 6, 8, 9 showed more significant vibrations in the region of 1170–1070 cm^−1^ (*ν*(NN)), indicating participation of the imine group in complexation. Strong absorption peaks were observed in the pure ligand at 1475–1449 cm^−1^, corresponding to the *ν*(C=C) vibration mode. These peaks shifted towards higher wavenumbers after complex formation. At lower wavelengths, bands were observed at 1406–1361, 998–949 and 970–690 cm^−1^ that correspond to *β*(CH), *δ*(C=C), and *γ*(CH), respectively.

Based on the obtained data, coordination in complexes 1, 2, 3, 5, and 7 is proposed *via* the nitrogen atom of the pyridine/pyrazine ring. For the other complexes, the imine nitrogen atom is involved in coordination. In these cases, ligands act as bidentants, chelating metal centers. Complex 4 is an exception, as the ligand forms an infinite, one-dimensional polymer chain upon binding to the metal, which is considered in the crystal structure part. The proposed coordination in complex 6 has been confirmed by the crystal structure of the compound. Spectra of ^1^H and ^13^C (Fig. S2–S5†) were also obtained for complex 7, which also (together with EA and F-AAS) clarify the proposed complex structure.

### NMR

The ^1^H NMR spectrum of the free ligand L_2_ (Fig. S2†) reveals several characteristic signals, including NH_2_ protons (at *δ* 7.16 and 7.48 ppm), pyridine protons (between *δ* 7.66 and 8.24), and a distinct azomethine proton at *δ* 8.57. Upon complexation with cadmium (complex 7), there are slight downfield shifts observed for the pyridine protons, indicating pyridine involvement in metal coordination (Fig. S3†). The ^13^C NMR (Fig. S4†) spectrum of L_2_ reveals characteristic signals from its pyridine and 4-nitrophenyl groups, connected through a hydrazone bond. Key signals are seen at *δ* 157.232 (C–Cl), 151.534 (C=N pyridine), and 142.103 ppm (C=N hydrazone). Upon complexation with Cd(ii), forming complex 7 (Cd(L_2_)_2_Cl_2_), there are specific downfield shifts in the pyridine ring carbon atoms (*e.g.*, *δ* 157.232 → 157.52 ppm), confirming the coordination through the pyridine nitrogen atoms (Fig. S5†). These changes in chemical shifts reflect the decreased electron density around the coordination sites, as electrons are transferred to the Cd(ii) center. The consistent spectral pattern in the aromatic/heterocyclic region (120–160 ppm) confirms that the structural framework of the ligand remains intact after coordination, without any degradation or rearrangement. This indicates successful synthesis of a stable and pure complex.

The tetrahedral geometry of the complex is supported by spectral changes in both ^1^H and ^13^C NMR spectra, where two L_2_ ligands are coordinated to cadmium *via* pyridine nitrogens, while two chloride ions complete the coordination sphere.

### Crystal structure

#### Complex 4

**Fig. 2:**
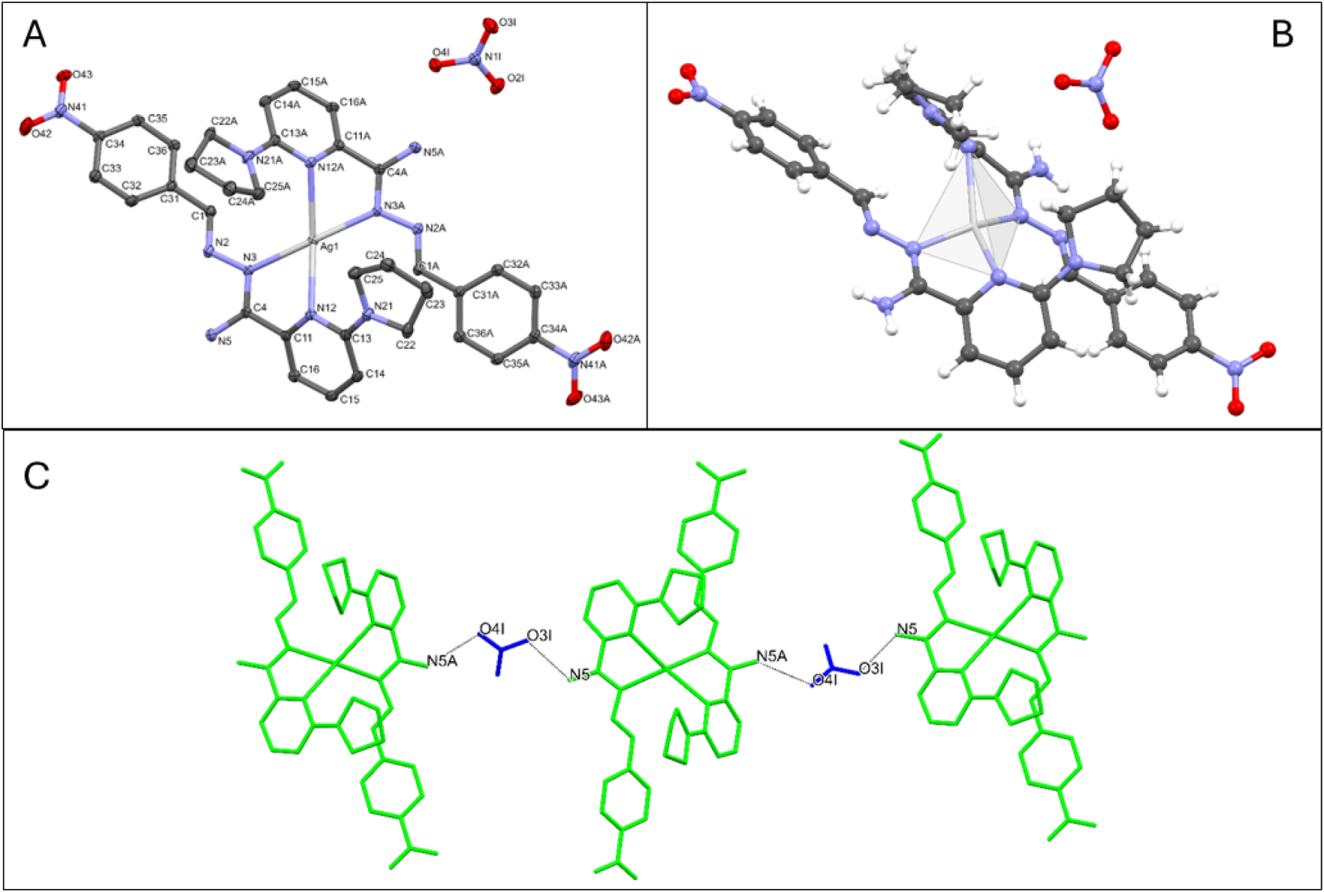
(A) Molecular structure of complex 4 showing atom-labelling schemes. Displacement ellipsoids are drawn at the 50% probability level. Hydrogen atoms have been removed for clarity. (B) Coordination polyhedral for complex 4. (C) The intermolecular hydrogen bonds in complex 4.

Crystal data, data collection, and structure refinement details are summarized in Table S2.† The coordination compound of cationic silver(i), containing two ligands and one silver nitrate salt [Ag(L_4_)_2_]NO_3_, is shown in Fig. 2A. The Ag atom is coordinated by four N atoms from two ligands, forming a distorted tetrahedral geometry, as shown in Fig. 2B (Table S3†). The nitrate ion forms an infinite chain through hydrogen bonds with NH_2_ groups of ligands (N–H⋯O), acting as a bridge in the chain, as depicted in Fig. 2C.

#### Complex 6

**Fig. 3:**
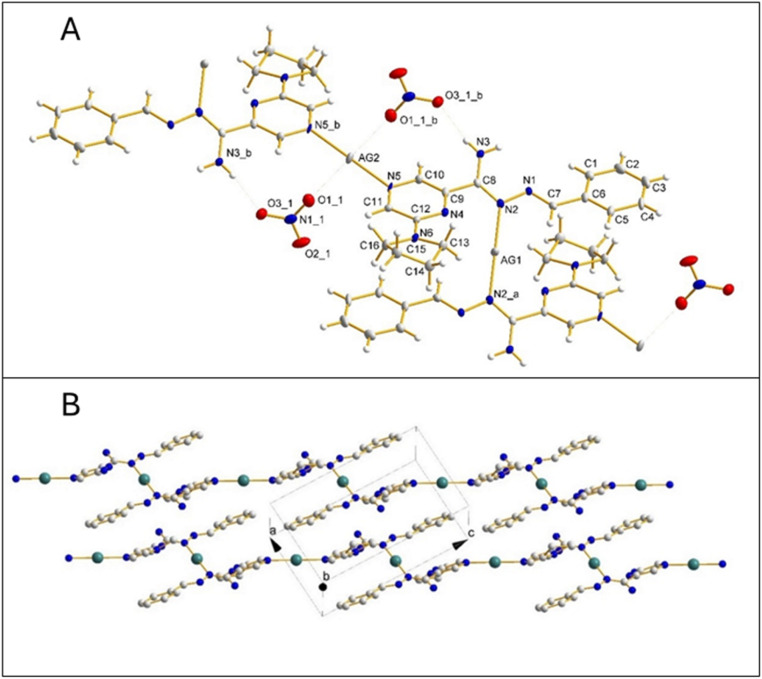
(A) Molecular structure of complex 6 with atom-labelling scheme. Displacement ellipsoids are drawn at the 50% probability level. Symmetry transformations used to generate equivalent atoms: suffix _*a* (−*x* + 2, −*y* + 1, −*z* + 1), suffix _*b* (−*x* + 1, −*y* + 1, -*z* + 2); (B) the crystal packing for complex 6. The nitrate anions and hydrogen atoms are omitted for clarity.

**Fig. 4:**
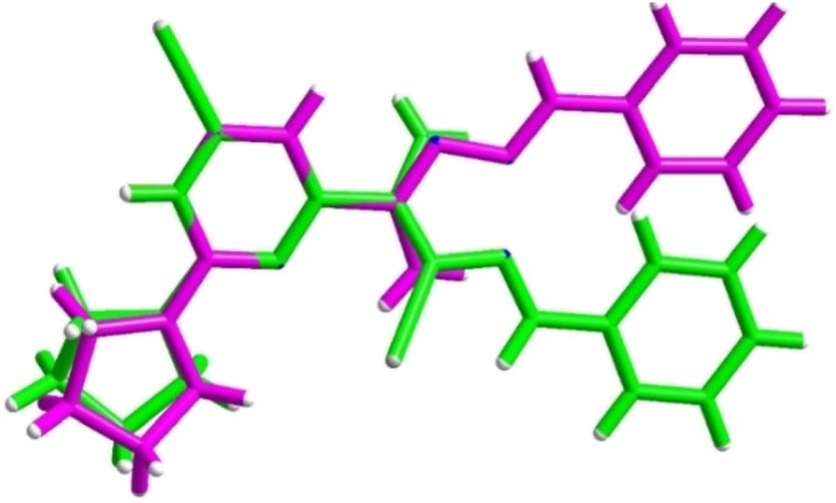
Superimposition of structures of the studied complex 6 (green) and the hydrated ligand molecule (CCDC 2241574, magenta).

Crystal data and final agreement parameters of complex 6 are provided in Tables S5–S7.† The asymmetric unit of the unit cell contains one molecule of ligand L_6_ (refs. 28 and 29) (CCDC 2241574) coordinated by two Ag atoms at two special positions with occupation ½ and one nitrate anion (Fig. 3A). Both Ag atoms are bonded to nitrogen atoms from two different but symmetrically related molecules of the ligand, forming an infinite one-dimensional polymer chain in the crystal structure (Fig. 3B). The incorporation of the ligand into the chain also changes the conformation of the ligand as can be seen from the significantly different values −32.7(6)° and 177.68(9)° of the torsion angle N2–C8–C9–N4 and N3–C4–C21–N26, here and in CCDC 2241574, respectively (Fig. 4).

### TGA

Fig. S6–S8† present thermal decomposition patterns of the obtained Ag(i) and Cd(ii) coordination compounds. Table 1 demonstrates the decomposition stages of the complexes with the identified temperature ranges. All investigated compounds were stable at room temperature and decomposed gradually during the heating process.

**Table 1:** Thermal decomposition data of complexes 1–9

Compound	Stages	Temperature ranges (°C)				Final product
		I	II	III	IV	
Complex 1	4	118–156	156–205	205–642	642–820	Ag
Complex 2	4	100–210	210–220	220–665	665–820	Ag
Complex 3	4	110–155	155–255	255–675	675–820	Ag
Complex 4	3	135–240	240–350	350–525	—	Ag
Complex 5	4	35–175	175–250	250–600	600–710	Ag
Complex 6	3	195–245	245–485	485–560	—	Ag
Complex 7	3	220–350	350–660	660–745	—	CdO
Complex 8	3	305–400	400–590	590–710	—	CdO
Complex 9	3	260–360	360–680	680–760	—	CdO

The first three complexes are stable up to 100 °C and more and decomposed gradually in a similar way. They consist 4 main decomposition steps. The first stage of decomposition of the complex 1 proves the presence of H_2_O molecule in these compounds (−3.84%). Complex 2 and 3 showed a low mass loss (less than 1%) of water from the surface of the test compounds. As the temperature increases, all three complexes undergo gradual thermal decomposition combined with the destruction of the organic ligand. The last stage for all coordination compounds is the decomposition of the residual parts of the organic ligand, loss of nitrate anions and the formation of Ag^34,35^ (22.41% for complex 1, 21.1% for complex 2, 24.3% for complex 3).

Complex 5 is stable at room temperature, but above 35 °C the dehydration process begins, which is the first step of decomposition. In the second step of thermal decomposition, destruction of morpholine is observed with a mass loss of 17.7% (theor. 17.5%). The process was completed in the fourth step at 710 °C, where the final solid product of decomposition is metallic Ag (21.2%).

Complexes 4 and 6 are the most stable among all studied silver complexes. Thermal decomposition patterns of these complexes contain three decomposition steps each in a similar manner. All compounds decompose progressively. The decompositions started at 135 °C and 185 °C with mass losses of 13.2% and 15.7%, corresponding to the combustion of pyrrolidine (complex 4, 13.8% theor.) and pyrrole (complex 6, 15% theor.). The last third step for these two coordination compounds is the decomposition of the residual parts of the organic ligand, loss of nitrate anions and the formation of Ag (19% for complex 4 and 23.4% for complex 6).

Complexes 7, 8, 9 demonstrate very high stability up to 220 °C, 305 °C, 260 °C, respectively. Decomposition started with the partial loss of part of organic ligands for all three complexes. The second mass losses can be assigned to the release of the rest parts of the organic ligands to form CdO. The total mass loss observed experimentally during the 3rd step at 710 °C and higher (96.18% for complexes 7, 95.65% for complexes 8, 96.52% for complexes 9). This behaviour can be connected with the CdCl_2_ volatilization.^36–38^

### Solution stability

**Fig. 5:**
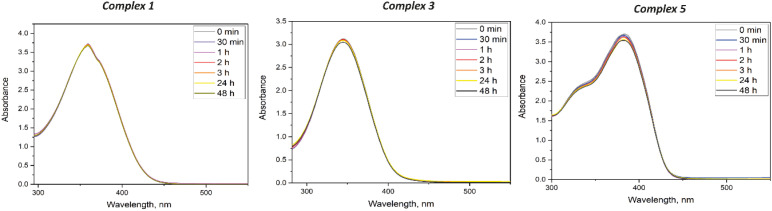
Time-dependant stability study of the complexes in DMSO.

The stability of metal complexes is a crucial property that provides insight into their interactions with biological molecules, as it directly impacts their reactivity, bioavailability, and effectiveness in therapeutic applications. Additionally, the solubility of these complexes in dimethyl sulfoxide (DMSO) is of considerable importance, as solubility data are essential for optimizing storage conditions and ensuring compatibility with biological assays.^39^ DMSO, a widely used solvent in drug discovery, serves as a medium that supports the stability and bioavailability of complexes under assay conditions, which is critical for maintaining the integrity of analytical protocols and enabling accurate assessments of biological activity. For the obtained complexes, the time-dependent UV-vis investigations in DMSO (Fig. 5 and S9†) were carried out from 0 min to 48 h (0 min, 30 min, 1 h, 2 h, 3 h, 24 h, 48 h).

The most stable compounds were complexes 1 and 3, where the hypochromic effect appeared to the least extent, representing 0.84% and 2.31%, respectively. At the same time hypsochromic shifts are absent. Similarly, no distinctive changes were observed for complexes 2, 4, 5, 6, and 9, where Δ*ε* ranges from 5% to 9.8%. Complexes 7 and 8 showed the least stable behaviour, where the hypochromic effect was 27.7% and 36.3%, and the peak maxima shifted by 15 and 4 nm, respectively.

### Antibacterial study

**Fig. 6:**
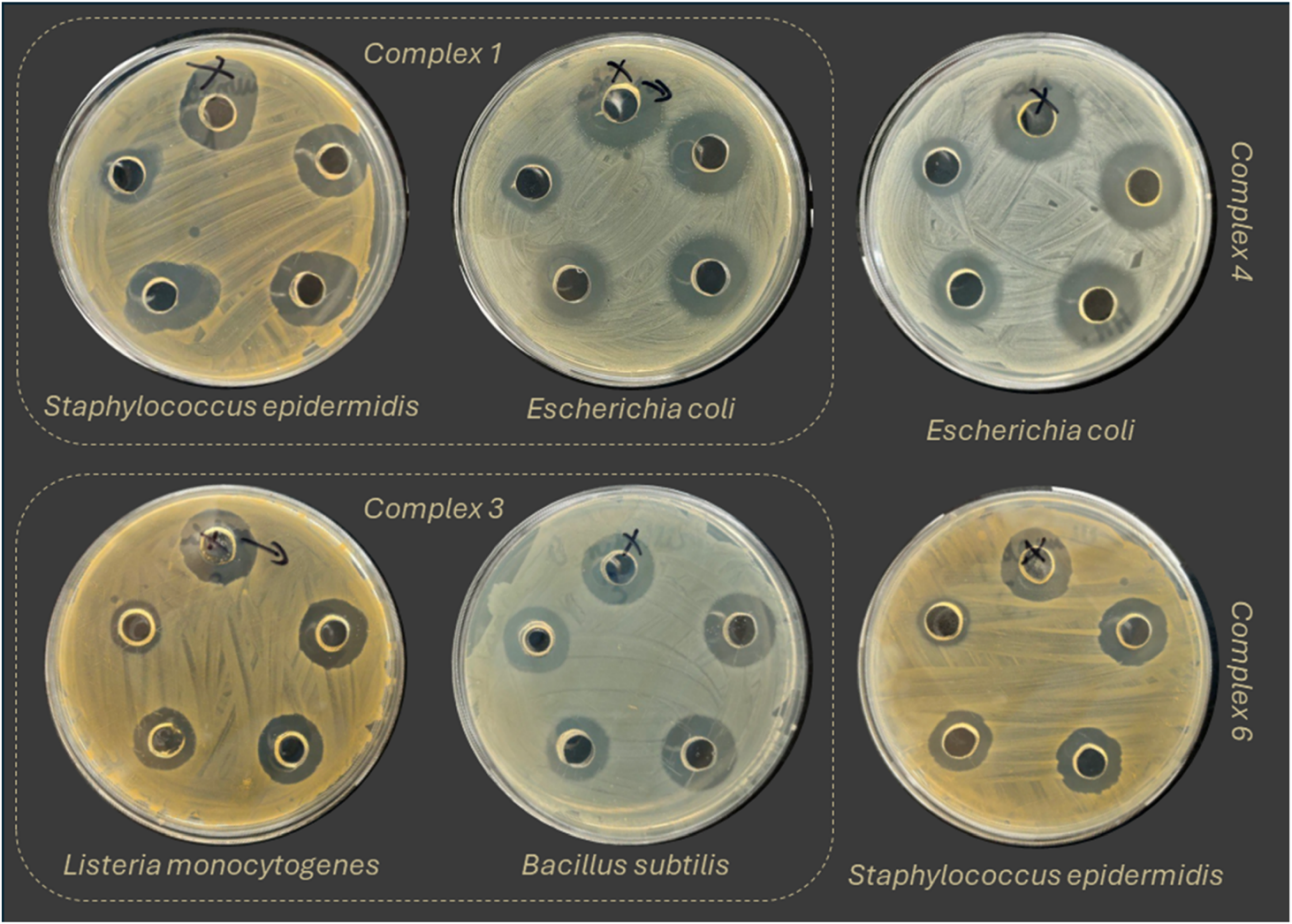
The inhibition zones of the complexes 1, 3, 4, 6.

In this study, the antibacterial activity of the Ag(i) and Cd(ii) complexes was investigated. The results were compared to the previous study, where antibacterial properties of their ligands were measured.^29^ Some of the results are presented in the Table 2, the whole data is available in the Table S8.† The inhibition zones around the wells (Fig. 6) were measured as well and presented in the Table S9.†

**Table 2:** The minimum inhibitory concentration (MIC) of the some investigated complexes inhibited the growth of the tested microorganisms (compared to its pure ligands)

Microorganism	Complexes and ligands								Ref. drug
	1	L_1_	4	L_4_	5	L_5_	6	L_6_	
Gram-positive bacteria	MIC (μg mL^−1^)								Van
*S. aureus* ATCC 25923	<10	500	<10	>500	50	>500	50	500	1
*S. epidermidis* ATCC 12228	10	500	50	>500	<10	>500	<10	500	1
*E. faecalis* ATCC 29212	50	250	50	>500	50	>500	>500	500	1
Gram-negative bacteria	MIC (μg mL^−1^)								Cip
*S. typhimurium* ATCC 14028	<10	>500	100	>500	50	>500	50	500	1

Complex 1 exhibited antibacterial activity against all tested microorganisms, showing MIC values <10 μg mL^−1^ for *Staphylococcus aureus*, *Listeria monocytogenes*, *Escherichia coli* and *Salmonella typhimurium*. Among all the tested complexes, complex 1 exhibited the strongest antibacterial activity. Complex 2 also exhibited activity against all tested microorganisms, except *Staphylococcus epidermidis*. The lowest MIC values (10 mg L^−1^) for complex 2 were obtained against *Listeria monocytogenes* and *Escherichia coli*. For complex 3 the results were similar, but MIC value of 10 μg mL^−1^ was also found for *Bacillus subtilis*. Complex 4 showed high antibacterial activity against *Staphylococcus aureus*, *Listeria monocytogenes*, *Escherichia coli* (MIC 10 or <10 μg mL^−1^). In the case of complex 5, it was determined that the complex showed high activity against all tested microorganisms except *Escherichia coli*. Among all tested microorganisms, complex 6 was not effective against *Enterococcus faecalis* and *Escherichia coli*. Its highest activity was against *Staphylococcus epidermidis* (MIC <10 μg mL^−1^). Compared to the silver complexes, the cadmium complexes exhibited lower antibacterial properties. However, in contrast to the corresponding ligands, complexes 8 and 9 exhibited moderate activity against both Gram-positive and Gram-negative bacteria. Among them, complex 9 exhibited the strongest antibacterial properties against *Staphylococcus aureus*, *Enterococcus faecalis*, *Escherichia coli* (10 μg mL^−1^). Complex 7 exhibited the lowest antibacterial activity among all tested compounds.

### Anticancer study

The MTT assay results after 48 hours of incubation demonstrated variable cytotoxic effects of the tested compounds across three cell lines: glioblastoma U87, LN-229, and normal fibroblast BJ (Table 3). Compounds L_1_, L_3_, L_5_ exhibited limited cytotoxicity with IC_50_ values exceeding 50 μM. However, significant cytotoxic activity was observed in certain metal complexes. For instance, complex 1 showed potent cytotoxicity in both LN-229 (IC_50_ = 25.22 ± 3.75 μM) and BJ cells (IC_50_ = 20.48 ± 5.21 μM). L_2_ and its Ag derivative (complex 2) also demonstrated activity, particularly in LN-229 (IC_50_ = 40.77 ± 6.12 μM and 37.24 ± 6.55 μM, respectively) and BJ cells (IC_50_ = 35.48 ± 4.33 μM and 14.46 ± 2.82 μM, respectively). Complex 4 showed the most significant cytotoxicity in U87 cells (IC_50_ = 1.22 ± 0.34 μM), while complex 9 was highly active in BJ cells (IC_50_ = 1.05 ± 0.32 μM). Interestingly, some compounds exhibited selective cytotoxicity. As an example, complex 6 showed strong activity across all three cell lines, with IC_50_ values of 10.17 ± 2.98 μM in U87, 20.98 ± 3.07 μM in LN-229, and 10.62 ± 2.14 μM in BJ cells. Overall, metal complexes of Ag(i) and Cd(ii) derivatives, generally exhibited greater cytotoxic potential compared to their parent ligands, with variations in cell line sensitivity. BJ fibroblasts were more susceptible, suggesting that metal complexation may enhance cytotoxic effects.

**Table 3:** IC_50_ values of the tested compounds obtained from the MTT assay after 48 hours of incubation. The data represent the concentration of compounds required to inhibit cell viability by 50%, indicating their cytotoxic potency. IC_50_ in μM

	U87	LN-229	BJ
L_1_	>50	>50	>50
Complex 1	8.15 ± 2.67	25.22 ± 3.75	20.48 ± 5.21
L_2_	>50	40.77 ± 6.12	35.48 ± 4.33
Complex 2	>50	37.24 ± 6.55	14.46 ± 2.82
Complex 7	>50	>50	41.66 ± 4.18
L_3_	>50	>50	>50
Complex 3	32.03 ± 7.08	>50	>50
Complex 8	10.87 ± 3.80	>50	25.84 ± 4.48
L_4_	>50	48.11 ± 6.09	>50
Complex 4	1.22 ± 0.34	>50	>50
Complex 9	>50	45.34 ± 2.18	1.05 ± 0.32
L_5_	>50	>50	>50
Complex 5	49.67 ± 3.11	>50	>50
L_6_	>50	>50	42.36 ± 6.17
Complex 6	10.17 ± 2.98	20.98 ± 3.07	10.62 ± 2.14
Cisplatin	39.56 ± 1.13	29.09 ± 0.23	>50

Ag(i) complexes of L_3_, L_4_ and L_5_ are the most promising compounds, that demonstrated selective toxicity towards cancer cells while showing no effect on normal cells (Fig. S10†).

**Fig. 7:**
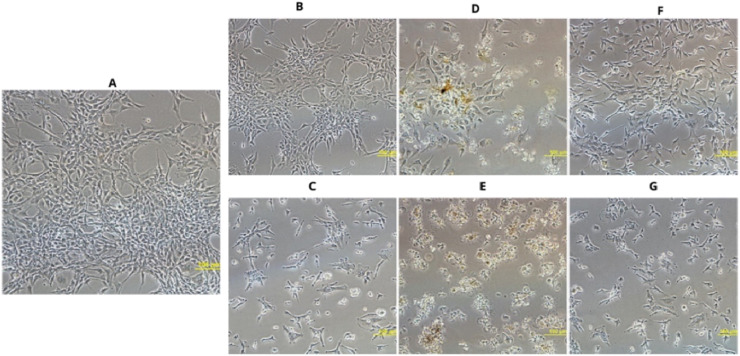
Morphology of U87 cells treated with L3, L4, L5 in concentration of 50 μM (B, D, F) and their Ag complexes 3, 4, 5 (C, E, G) in concentration corresponding to IC50 value (32.03, 1.82 and 49.67 μM respectively). (A) Control culture treated with DMSO as vehicle. Magnification 200.

Microscopic observations confirmed the results of the MTT assay. Cells treated with the Ag-containing complexes shrank, which is a sign of toxic effects (Fig. 7C, E and G). Cells exposed to the complex 4 appeared more rounded and detached from the substrate (Fig. 7E), indicating that this compound has the strongest effect. Ligands L_3_, and L_5_ did not exhibit such effects, and the cell morphology remained similar to that of normal cells (Fig. 7B and F). Incubation with the L_4_ ligand caused slight, partial cell shrinkage (Fig. 7D).

**Fig. 8:**
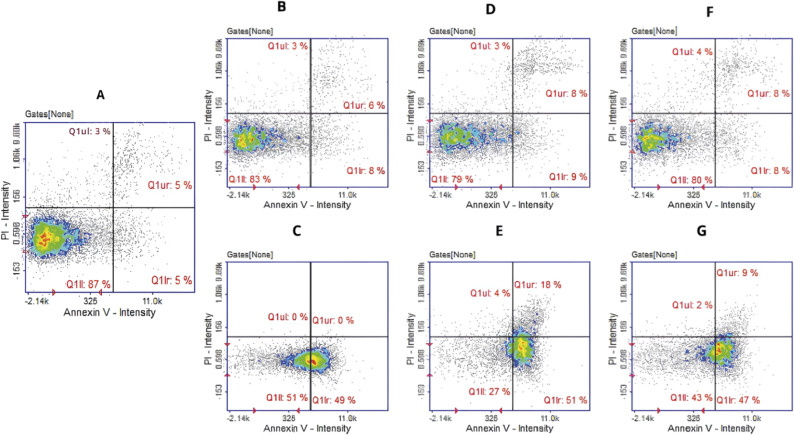
Apoptotic and necrotic cell detection by image cytometry. U87 cells were treated with L_3_, L_4_, L_5_ in concentration of 50 μM (B, D, F) and their Ag complexes 3, 4, 5 (C, E, G) in concentration corresponding to IC50 value (32.03, 1.82 and 49.67 μM respectively). (A) Control culture treated with DMSO as vehicle. The results show one representative experiment of three independently performed. Q1II—live, Q1Ir—early apoptotic, Q1ur—late apoptotic, and Q1uI—necrotic cells.

Apoptosis detection revealed that approximately 50% of the cells incubated with the complexes 3 and 5 were in the early stage of apoptosis (Fig. 8C and G). In the case of the complex 4, a population of cells in the late stage of apoptosis was also present, confirming the stronger effect of this compound (Fig. 8E). Histograms for cultures treated with the ligands did not differ significantly from the histograms for control cells (Fig. 8B, D and F).

**Fig. 9:**
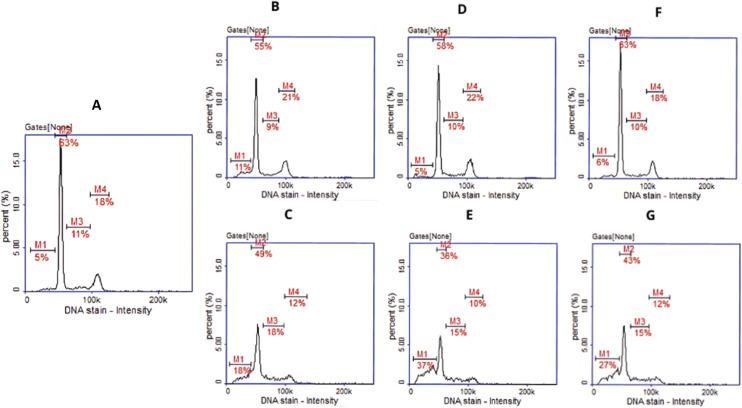
Cell cycle analysis by image cytometry. U87 cells were treated with L_3_, L_4_, L_5_ in concentration of 50 μM (B, D, F) and their Ag complexes 3, 4, 5 (C, E, G) in concentration corresponding to IC_50_ value (32.03, 1.82 and 49.67 μM respectively). (A) Control culture treated with DMSO as vehicle. The results show one representative experiment of three independently performed (M1 – subG1, M2 – G1, M3 – S, M4 – G2/M phase).

Cell cycle analysis showed that in cultures treated with Ag(i) complexes, the number of cells in the G1 and G2/M phases decreased, while the number of subG1 cells, which correspond to apoptotic cells, increased. This was most notable for complex 4, as shown in Fig. 9. No cell cycle arrest was observed, indicating that the complexes have a cytotoxic rather than a cytostatic effect.

## Experimental part

The chemical compounds for the complexes and ligands syntheses were obtained from Sigma-Aldrich. The chemicals were used without additional purification.

### Synthesis of the ligands

The synthesis routes, structures and properties of the ligands, used for this work, were fully described in our previous publications^28,29^ and presented in ESI.†

### Synthesis of the complexes

A suitable amount of ligand was dissolved in methanol, with constant stirring at 90 °C. After the ligands have been dissolved, a metal salt (AgNO_3_ or CdCl_2_), dissolved in water, was added to each solution in an equimolar ratio of 1 : 1. The mixtures were left to stir at 90 °C for two hours. After that, they were cooled to room temperature and stored in the refrigerator to allow the formation of complexes. The resulting precipitates were filtered, washed with methanol, and dried at room temperature.

### X-ray crystallographic analysis of complex 4

Diffraction measurements were performed using a XtaLAB Synergy, Dualflex diffractometer, (Rigaku Oxford Diffraction, Poland–Japan–UK), with a Pilatus 300 K detector at low temperature (100.0(2) K) using MoKα radiation (0.71073 Å). Diffraction data were processed using CrysAlisPRO software (Rigaku Oxford Diffraction, CrysAlisPRO; Rigaku Oxford Diffraction Ltd: Yarnton, Oxfordshire, England). Solving and refinement of the crystal structure was performed with SHELX^40,41^ using full-matrix least-squares minimization on F2. All H atoms were geometrically optimized and allowed as riding atoms. ShelXle and Mercury softwares^42,43^ were used to visualize the molecular structure.

CCDC 2403771† contains the supplementary crystallographic data for this paper. These data can be obtained free of charge from The Cambridge Crystallographic Data Centre *via*https://www.ccdc.cam.ac.uk/structures.

### X-ray crystallographic analysis of complex 6

Single crystal of complex 6 was obtained by one-spot synthetic method at temperature 90 °C. X-ray diffraction data were collected at 119.98(13) K with a Rigaku Oxford Diffraction Synergy Custom system and a Rigaku HyPix-6000HE detector using Mo Kα radiation (*λ* = 0.710 73 Å) generated by a Rigaku MicroMax-007 HF DW. The data collection, reduction, and absorption corrections were performed using CrysAlisPro (version 1.171.43.112a).^44^ The crystal structure was solved and refined using SHELXT.^41,45^ Images of the structure were created using Diamond 5.0.2.^46^ The Crystallographic Information File for complex 6 has been deposited in the Cambridge Crystallographic Data Centre (CCDC deposition number 2388390†), from which it can be obtained free of charge using https://www.ccdc.cam.ac.uk/structures/.

### F-AAS

The content of solid metals were determined using F-AAS (Flame Atomic Absorption Spectroscopy) spectroscopy with a source of light and using air/acetylene flame. Analytical spectral lines were used for absorbance measurement where the limit of quantification was 0.04 mg L^−1^. Solid samples were decomposed using the Anton Paar Multiwave 3000 closed system instrument. Mineralization conditions: 240 °C under pressure 60 bar for 45 min.

### Elemental analysis

The contents of carbon, hydrogen and nitrogen were determined by a Vario micro company Elementar Analysensysteme GmbH.

### FTIR

FTIR spectra were recorded with an IRTracer-100 Shimadzu Spectrometer (4000–600 cm^−1^) with the accuracy of recording 1 cm^−1^ using KBr pellets.

### Thermogravimetric analysis

The thermal analyses were carried out with a Netzsch STA 449 F1 Jupiter thermoanalyzer (Netzsch-Geratebau GmbH, Selb, Germany) coupled with a Netzsch Aeolos Quadro QMS 403 mass spectrometer (Netzsch-Geratebau GmbH, Selb, Germany). The thermolysis of compounds in the air atmosphere was studied by TG-DTA techniques in the range of temperature 25–800 °C at a heating rate of 10 °C min^−1^; TGA-MS and DTA curves were recorded under the air atmosphere *v* = 20 mL min^−1^ using ceramic crucibles, and as a reference material ceramic crucible was used.

### Solution stability

The sample solutions (50 μL (1.0 × 10^−3^ M)) of the complexes were prepared in DMSO (Sigma Aldrich, ≥99.9%). The absorption spectra of the complexes were measured with increasing time (0 min, 30 min, 60 min, 2 h, 3 h, 24 h, 48 h) at room temperature. All measurements were performed on the Shimadzu UV-2401PC.

### Antibacterial study

#### Determination of antibacterial activity

The antibacterial activity of tested samples was assessed against both gram-positive bacteria (*Staphylococcus aureus* ATCC 25923, *Staphylococcus aureus* ATCC 6538, *Staphylococcus epidermidis* ATCC 12228, *Enterococcus faecalis* ATCC 29212, *Listeria monocytogenes* ATCC 19115, *Bacillus subtilis* ATCC 6633, *Bacillus cereus* ATCC 10876) and gram-negative bacteria (*Escherichia coli* ATCC 10530, *Salmonella typhimurium* ATCC 14028). Antibacterial properties were evaluated using both the dilution method (employing DMSO and 96-well plates) and the diffusion-well method (using Mueller Hinton Agar, Merck, Germany).

24 hour bacterial cultures with a density of 1–2 × 10^8^ CFU mL^−1^ were plated (0.1 mL) on Mueller–Hinton Agar (Merck, Germany). Subsequently, wells with a diameter of 10 mm were cut out from and filled with 100 μL of the tested compound at a concentration of 10–500 μg mL^−1^ (diluted in DMSO). After an 18-hour incubation at either 30 °C (for *Bacillus* ssp.) or 37 °C (for other tested microorganisms), the inhibition zones around the wells were measured, and the results were expressed in millimetres (Table S9†).

#### Minimum inhibitory concentration

The minimum inhibitory concentrations (MICs) of the tested samples were evaluated against a panel of reference microorganisms obtained from the American Type Culture Collection (ATCC), including Gram-negative bacteria (*Escherichia coli* ATCC 10530, *Salmonella typhimurium* ATCC 14028), and Gram-positive bacteria (*Staphylococcus aureus* ATCC 25923, *Staphylococcus aureus* ATCC 6538, *Staphylococcus epidermidis* ATCC 12228, *Enterococcus faecalis* ATCC 29212, *Listeria monocytogenes* ATCC 19115, *Bacillus subtilis* ATCC 6633), and one strain (*Bacillus cereus* ŁOCK 0807) obtained from the Pure Culture of Industrial Microorganisms of the Institute of Fermentation Technology and Microbiology ŁOCK 105 (Łódź, Poland).

The microorganism cultures with a density of 1.5 × 10^8^ CFU mL^−1^ (corresponding to a McFarland standard of 0.5) were plated on Mueller–Hinton Agar (Merck, Germany) following the procedures recommended by the European Committee on Antimicrobial Susceptibility Testing (EUCAST).^47^ Wells measuring 10 mm in diameter were cut into the medium. The tested compounds were dissolved in a DMSO solution, which was recommended due to their chemical structures. Subsequently, 100 μL of prepared solutions containing the tested compounds at concentrations ranging from *x* μg mL^−1^ to *x* μg mL^−1^ were added to each well. The plates were incubated at 30 °C (for *Bacillus subtilis* and *Bacillus cereus*) or 37 °C (for other bacteria) for 18 hours. After incubation, the minimum inhibitory concentration (MIC value) was determined (the lowest concentration of the tested compounds that prevents bacterial growth) and expressed in μg mL^−1^. The experiment was conducted in three independent replicates. Ciprofloxacin (Sigma Aldrich, Saint Louis, MO, USA) was used as a reference antimicrobial agent against Gram-negative bacteria, while vancomycin (Sigma Aldrich, Saint Louis, MO, USA) was used as a reference antimicrobial agent against Gram-positive bacteria.

### Anticancer study

#### Cell culture

The glioblastoma cell lines LN-229 and U87 and the reference human normal skin fibroblast BJ were obtained from the American Type Culture Collection (ATCC). Cells were grown as monolayers when cultured in cell culture flasks and cultured in Dulbecco's Modified Eagle Medium (DMEM) – LN-229 and Eagle's Minimum Essential Medium (EMEM) – U87 and BJ, supplemented with 10% (v/v) fetal bovine serum, penicillin (10 000 U mL^−1^), streptomycin (10 mg mL^−1^) and amphotericin B (250 μg mL^−1^). The cells were incubated at 37 °C, 5% CO_2_ atmosphere. The cells were maintained in the logarithmic growth phase by regular passage at 80% confluence.

#### Cell viability assay

The cytotoxicity of the tested compounds was evaluated against both human glioblastoma LN-229 and U-87 as well as normal BJ cell lines. The cells were plated in 96-well culture plates (2 × 10^4^ well) and after 24 h incubation the cells were treated with the tested compounds in the concentration range 1–50 μM. The cells were cultured for the next 48 h. The cytotoxicity of the compounds was evaluated using the MTT colorimetric method as previously described.^28,29^ IC_50_ values were determined using the AAT Bioquest IC_50_ calculator as the concentration of compounds capable of reducing cell viability by 50%. The experiment was performed in triplicate for each concentration. To examine the cytotoxic effect of compounds on cell morphology, cells were examined under an inverted phase contrast microscope Nikon Eclipse Ti with NIS Element software (Tokyo, Japan).

#### Cell cycle assay

Cell cycle analysis was performed using the NucleoCounter® NC-3000™ image cytometry system (ChemoMetec USA Inc., Lillerød, Denmark). U-87 cells were seeded in 6-well plates at a density of 20 × 10^4^ mL 24 hours before the experiment. The cells were then treated with complexes 3, 4, 5 at concentrations corresponding to the IC_50_ value and ligands at 50 μM. Control cultures were treated with DMSO as vehicle. Cell cycle analysis was performed after 48 hours according to the manufacturer's protocol for two-step cell cycle analysis as previously described.^28,29^

#### Cell apoptosis assay

Apoptotic and necrotic cells were detected using the NucleoCounter® NC-3000™ image cytometry system (ChemoMetec USA Inc., Lillerød, Denmark). U-87 cells were seeded in 6-well plates at a density of 20 × 10^4^ mL^−1^ 24 hours before the experiment. The cells were then treated with complexes 3, 4, 5 at concentrations corresponding to the IC_50_ value and ligands at 50 μM. Control cultures were treated with DMSO as vehicle. Analysis was performed after 48 hours according to the manufacturer's protocol for the Annexin V assay as previously described.^28,29^

## Conclusion

In this study nine new Ag(i) and Cd(ii) coordination compounds with the hydrozanomide-based ligands were synthesized and characterized as potential antibacterial and anticancer agents. The newly obtained complexes were characterized using numerous physicochemical and spectral methods of analysis. The crystal structure for complexes 4 and 6 was determined, where complex 4 is a coordination compound of cationic silver(i) with two ligands and one silver nitrate salt, forming a distorted tetrahedral arrangement for Ag(i). In the case of complex 6, the metal atoms link molecules of the ligand in the crystal structure into an one-dimensional chain, and the asymmetric cell contains one molecule of the ligand coordinated by 2 Ag atoms in two special positions with occupation ½ and one nitrate anion. In the case of the other coordination compounds, based on the difference in the FTIR spectra of the complexes and free ligands, the coordination between metal–organic ligand takes place. The thermal decomposition patterns of the Ag(i) and Cd(ii) coordination compounds exhibit varying stability and decomposition behaviours. While all compounds are stable at room temperature, their decomposition occurs in multiple stages, involving the loss of water, organic ligands, and nitrate anions, ultimately forming metallic Ag or CdO. Complexes 4 and 6 exhibit the highest stability among the silver complexes, while complexes 7, 8, and 9 show exceptional thermal stability, with decomposition involving organic ligand loss and CdO formation, followed by potential CdCl_2_ volatilization at high temperatures. Besides that, a study has been carried out to examine the stability of complexes upon 48 h in a DMSO, where the most stable compounds were complexes 1 and 3.

The results of the antibacterial activity of complexes were compared to the activity of the parent ligands, which was measured in the previous study. All tested compounds demonstrated high or moderate activity against the studied microorganisms, specifically against *Staphylococcus aureus* and *Escherichia coli.* Complexes 1 and 4 showed the most promising activity, where MIC values were 10 μg mL^−1^ or <10 μg mL^−1^ against some of the Gram-negative and Gram-positive bacteria. Based on the achieved results, it was found that despite the overall higher toxicity of cadmium compared to silver, the synthesised silver complexes showed more potential as antibacterial agents.

The IC_50_ results demonstrated, that certain metal complexes exhibited significant cytotoxic activity. Complexes 1, 6, 8 showed high toxicity values towards the tested glioblastoma cells. At the same time, they also showed high toxicity to normal BJ cells, which makes it difficult to consider them as potential drug candidates. The most promising compounds for further research are those that exhibit selective toxicity towards cancer cells without harming normal cells, as has been shown by complexes 3, 4 and 5. Under the toxic influence of these complexes, the cells shrank, which was confirmed by microscopic observations. Based on the cell apoptosis assay results, it can clearly be observed that in the case of complex 4, a population of cells at a late stage of apoptosis was present, indicating a stronger effect of this compound. These findings confirm, that complex 4 can be considered as a potential selective anticancer metallodrug candidate and can be subjected to further biological studies.

## Author contributions

Conceptualization, A. C. (Alina Climova), A. C. (Agnieszka Czylkowska).; methodology, A. C. (Alina Climova), E. P., M. S., K. G., A. K.-P., M. I., E. K.-B., S. S., J. M., A. C. (Agnieszka Czylkowska); formal analysis, A. C. (Alina Climova), E. P., M. S., K. G., A. K.-P., M. I., J. M., A. C. (Agnieszka Czylkowska); investigation A. C. (Alina Climova), E. P., M. S., K. G., M. I., J. M.; data curation, A. C. (Alina Climova), A. C. (Agnieszka Czylkowska); writing – original draft preparation, A. C. (Alina Climova), E. P., M. S., A. K.-P., M. I., J. M., A. C. (Agnieszka Czylkowska); writing – review and editing, A. C. (Alina Climova), E. P., A. C. (Agnieszka Czylkowska); visualization, A. C. (Alina Climova); supervision, A. C. (Agnieszka Czylkowska). All authors have read and agreed to the published version of the manuscript.

## Conflicts of interest

There are no conflicts to declare.

## Supplementary Material

RA-015-D5RA02001H-s001

RA-015-D5RA02001H-s002

## Data Availability

Crystallographic data for complex 4 has been deposited at the https://www.ccdc.cam.ac.uk/structures with the deposition number CCDC 2403771; crystallographic data for complex 6 has been deposited at the https://www.ccdc.cam.ac.uk/structures with the deposition number CCDC 2388390; crystallographic data for L_6_ has been deposited at the https://www.ccdc.cam.ac.uk/structures under CCDC 2241574 and can be obtained from DOI: 10.5517/ccdc.csd.cc2f7jv2 (https://dx.doi.org/10.5517/ccdc.csd.cc2f7jv2); the data supporting this article have been included as part of the ESI.†
