# Supplementary material for: Synthesis of new Ag(i) and Cd(ii) hydrozanomide-based complexes with antibacterial and anticancer properties
Source: RSC Adv. 2025 Jun 10;15(25):19598–609. doi: 10.1039/d5ra02001h (PMC12150283; doi:10.1039/d5ra02001h)
Supplement: RA-015-D5RA02001H-s001 [file RA-015-D5RA02001H-s001.pdf]

Supplementary Information (SI) for RSC Advances.
This journal is © The Royal Society of Chemistry 2025

Synthesis of new Ag(I) and Cd(II) hydrozanomide-based complexes with antimicrobial and anticancer properties

Alina Climova[a*], Ekaterina Pivovarova[a], Małgorzata Szczesio[a], Katarzyna Gobis[b], Agnieszka Korga-Plewko[c], Magdalena Iwan[c], Edyta Kordialik-Bogacka[d], Sylwia Ścieszka[d], Jaromir Marek[e,f], Agnieszka Czylkowska[a*]

[a]Institute of General and Ecological Chemistry, Faculty of Chemistry, Lodz University of Technology, Zeromskiego 116, 90-924 Lodz, Poland

[b] Department of Organic Chemistry, Faculty of Pharmacy, Medical University of Gdańsk, 107 Gen. Hallera Ave., 80-416 Gdańsk, Poland

[c] Independent Medical Biology Unit, Faculty of Pharmacy, Medical University of Lublin, Jaczewskiego 8b, 20-093 Lublin, Poland

[d]Institute of Fermentation Technology and Microbiology, Faculty of Biotechnology and Food Sciences, Lodz University of Technology, Wólczańska 171/173, 90-924 Lodz, Poland

[e]Core Facility Biomolecular Interactions and Crystallography, CEITEC MU, Masaryk University, Kamenice 5, 62500 Brno, Czech Republic

[f]Department of Chemistry, Faculty of Science, Masaryk University, Kamenice 5, 62500 Brno, Czech Republic

*Corresponding authors: alina.climova@dokt.p.lodz.pl, agnieszka.czylkowska@p.lodz.pl

Table of content

Contents

Figure S1 – FTIR-spectra of complexes and corresponding ligands. 3

Table S1. The FTIR wavenumbers for ligands and complexes, cm^{-1}. 4

Figure S2 – ^1H NMR of L_2 (700 MHz, DMSO-d6): d 7.16, 7.48 (2s, 2H, NH2), 7.66 (d, 1H, pyridine), 7.98 (t, 1H, pyridine), 8.19-8.24 (m, 4H Ph + 1H pyridine), 8.57 (s, 1H, CH) ppm.5

Figure S3 – ^1H NMR of Complex 7 (700 MHz, DMSO-d6): d 7.16, 7.49 (2s, 2H, NH2), 7.67 (d, 1H, pyridine), 7.99 (t, 1H, pyridine), 8.21-8.29 (m, 4H Ph + 1H pyridine), 8.58 (s, 1H, CH) ppm. 6

Figure S4 – ^{13}C NMR of L_2 (175 MHz, DMSO-d6): d 121.145, 124.223 (2C), 126.679, 129.266 (2C), 141.121, 142.103, 148.292, 149.897, 151.534, 152.877, 157.232 ppm. 7

Figure S5 – ^{13}C NMR of Complex 7 (175 MHz, DMSO-d6): d 121.137, 124.208 (2C), 126.696, 129.275 (2C), 141.190, 142.111, 148.407, 150.034, 151.662, 153.044, 157.251 ppm. 7

Table S2. Experimental details. 8

Table S3. Geometric parameters (Å, °) for Ag compound. 9

Table S4. Hydrogen-bond geometry (Å, °) for **Ag** complex. 10

Table S5. Crystal data of complex 6. 11

Table S6. Bond distances [Å] and angles [°] for Ag atoms. Symmetry codes: a -x+2,-y+1,-z+1 ; b -x+1,-y+1,-z+2. 12

Table S7. Hydrogen-bond geometry [Å, °] for A. Symmetry code: b -x+1,-y+1,-z+2. 13

Figure S7 – TG (green) and DTG (blue) decomposition curves in air of the complexes 4, 5, 6. 15

Figure S8 – TG (green) and DTG (blue) decomposition curves in air of the complexes 7, 8, 9. 16

Figure S9 – Time-dependant stability study of the complexes in DMSO. 17

Table S8. Antimicrobial activity of tested complexes and reference drugs............................18

Table S9. Inhibition zones of the tested complexes in the concentration 500 μg/mL. 19

Figure S10 – Cell viability based on MTT test results. A, C, E – viability of normal fibroblast BJ treated with L_3, L_4, L_5 and their Ag complexes **3**, **4**, **5**; B, D, F – viability of glioblastoma U87 cells treated with L_3, L_4, L_5 and their Ag complexes 3, 4, 5. Control cultures (assumed to be 100%) were treated with DMSO as vehicle. 20

Synthesis of the ligands L_1-L_3 21

Synthesis of the ligands L_4-L_6 23

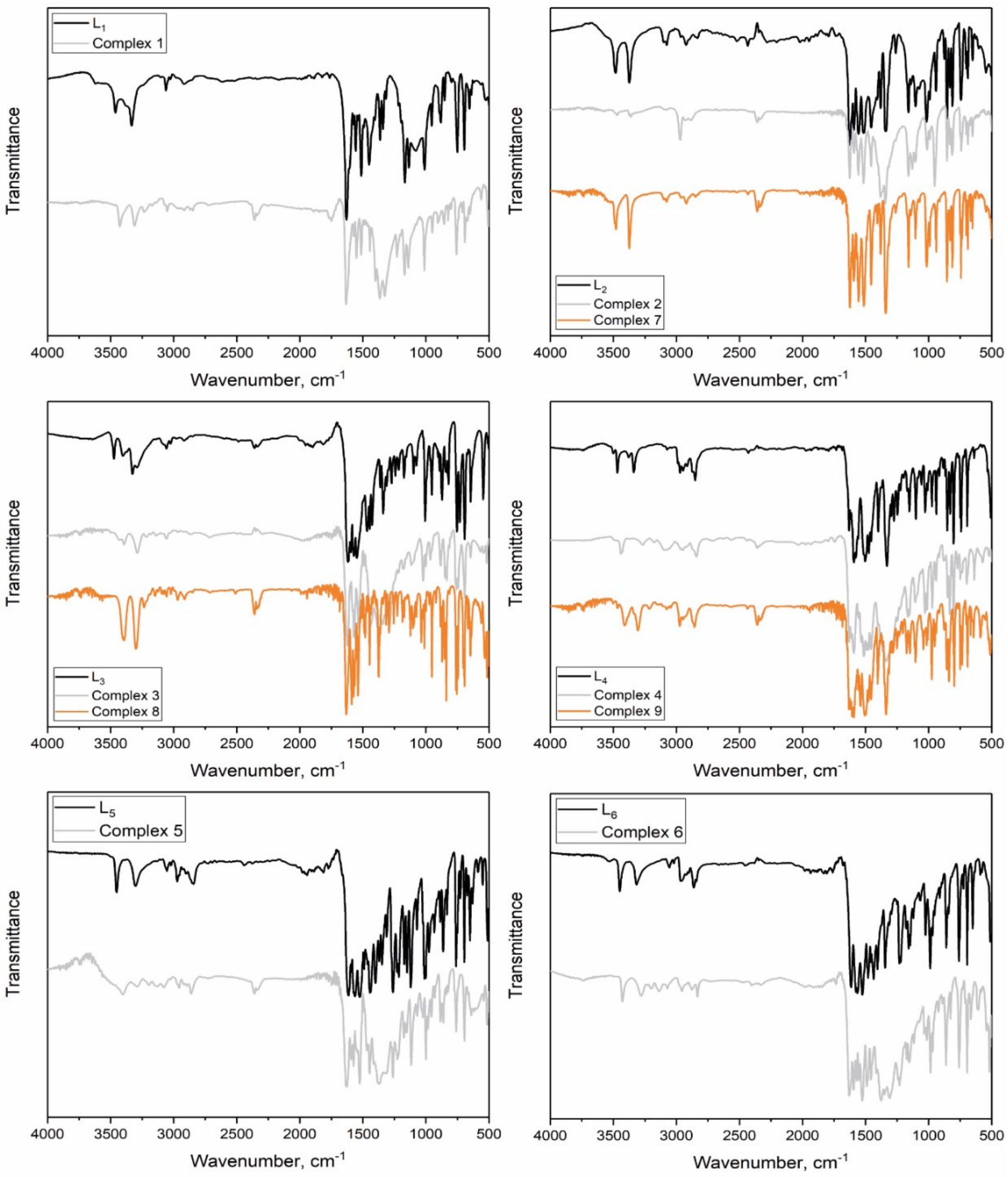

Figure S1 – FTIR-spectra of complexes and corresponding ligands.

Table S1. The FTIR wavenumbers for ligands and complexes, cm^{-1}.

	L_1	Complex 1	L_2	Complex 2	Complex 7	L_3	Complex 3	Complex 8	L_4	Complex 4	Complex 9	L_5	Complex 5	L_6	Complex 6
ν(NH)	3461<br>3332	3427<br>3311	3481<br>3373	3468<br>3361	3480<br>3374	3473<br>3292	3393<br>3288	3393<br>3300	3468<br>3339	3440<br>3267	3410<br>3306	3452<br>3304	3403<br>3288	3450<br>3317	3349<br>3279
ν(CH)	3060	3052	3078	3102	3107	3056	3057	3095	3074	3079	3078	3053	3080	3055	3063
ν(C=N)	1629	1632	1625	1626	1624	1616	1622	1632	1632	1631	1630	1614	1629	1614	1629
δ (NH)	1556	1550	1556	1558	1557	1548	1560	1538	1558	1593	1540	1562<br>1526	1573<br>1525	1578<br>1566	1527<br>1565
ν(C=C)	1450	1444	1456	1457	1456	1467	1445	1447	1469	1449	1457	1445<br>1489	1447	1479<br>1458	1490<br>1449
ν(NO2)	-	-	1515<br>1340	1571<br>1343	1594<br>1341	-	-	-	1331	1514<br>1338	1507<br>1338	-	-	-	-
β (CH)	1363	1366	1380	1382	1381	1361	1373	1376	1400	1360	1404	1371	1372	1406	1377
ν (CN)	-	1229	-	-	1262	1245	1270	1256<br>1228	1248<br>1275	1260	1251	1266	1262<br>1226	1232<br>1221	1235
ν (CO)	-	-	-	-	-	-	-	-	-	-	-	1215	-	-	-
ν (NN)	1166	1169	1161<br>1016	1160<br>1130	1160<br>1105	1174	1175<br>1120	1186<br>1123	1028	1165<br>1106<br>1029	1175<br>1149	1121	1117<br>1069	1024	1147<br>1024
δ(C=C)	955	949	991<br>941	998<br>952	995<br>940	953	1022	953	974	971	975	976	993	987	985
γ(CH)	881	855	854	854	854	871	881	870	856	881	857	864	885	860	862
ν (CCl)	696	691	744	688	689	692	693	648	-	-	-	-	-	-	-

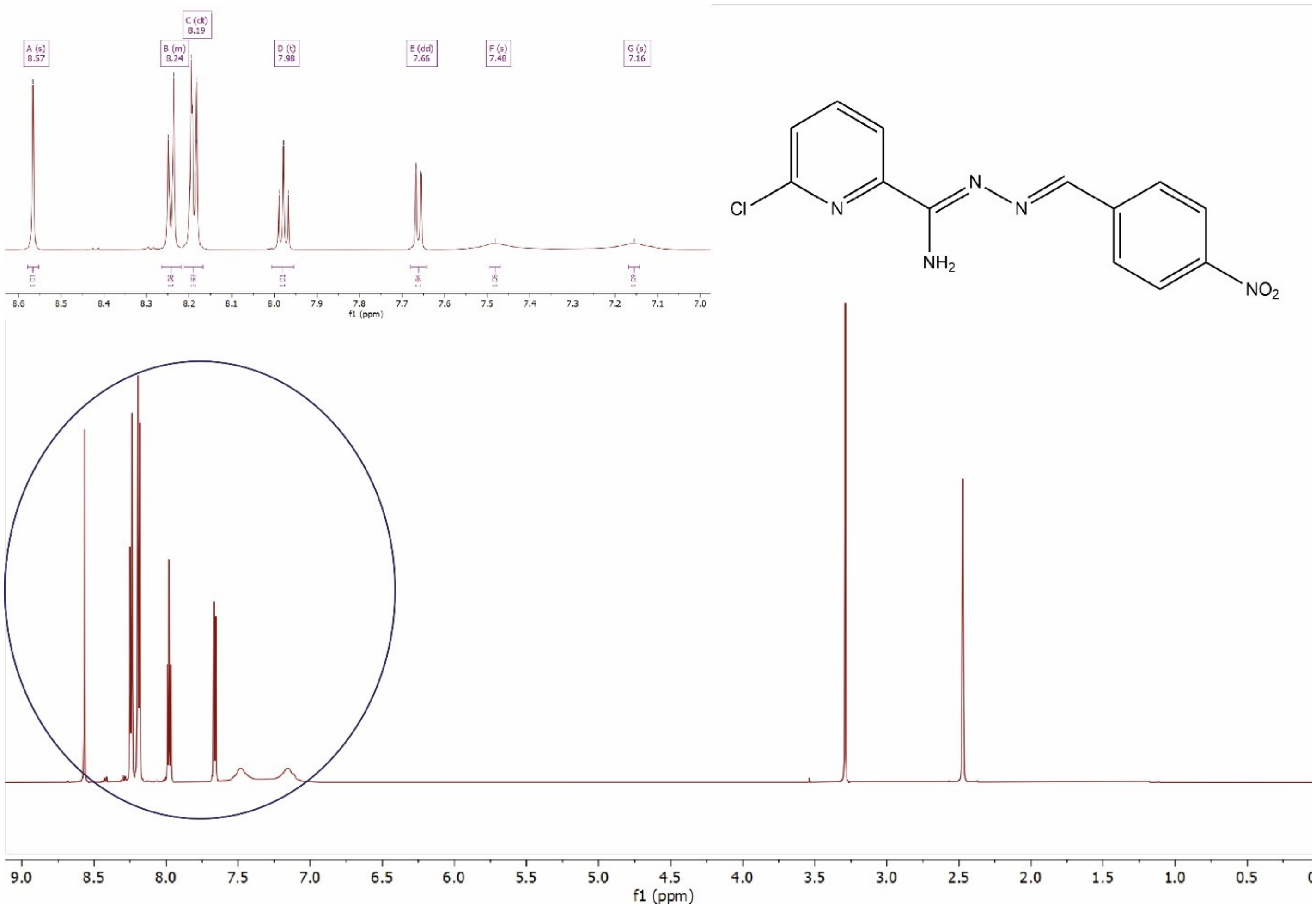

Figure S2 – ^{1}H NMR of L_2 (700 MHz, DMSO-d6): d 7.16, 7.48 (2s, 2H, NH2), 7.66 (d, 1H, pyridine), 7.98 (t, 1H, pyridine), 8.19-8.24 (m, 4H Ph + 1H pyridine), 8.57 (s, 1H, CH) ppm.

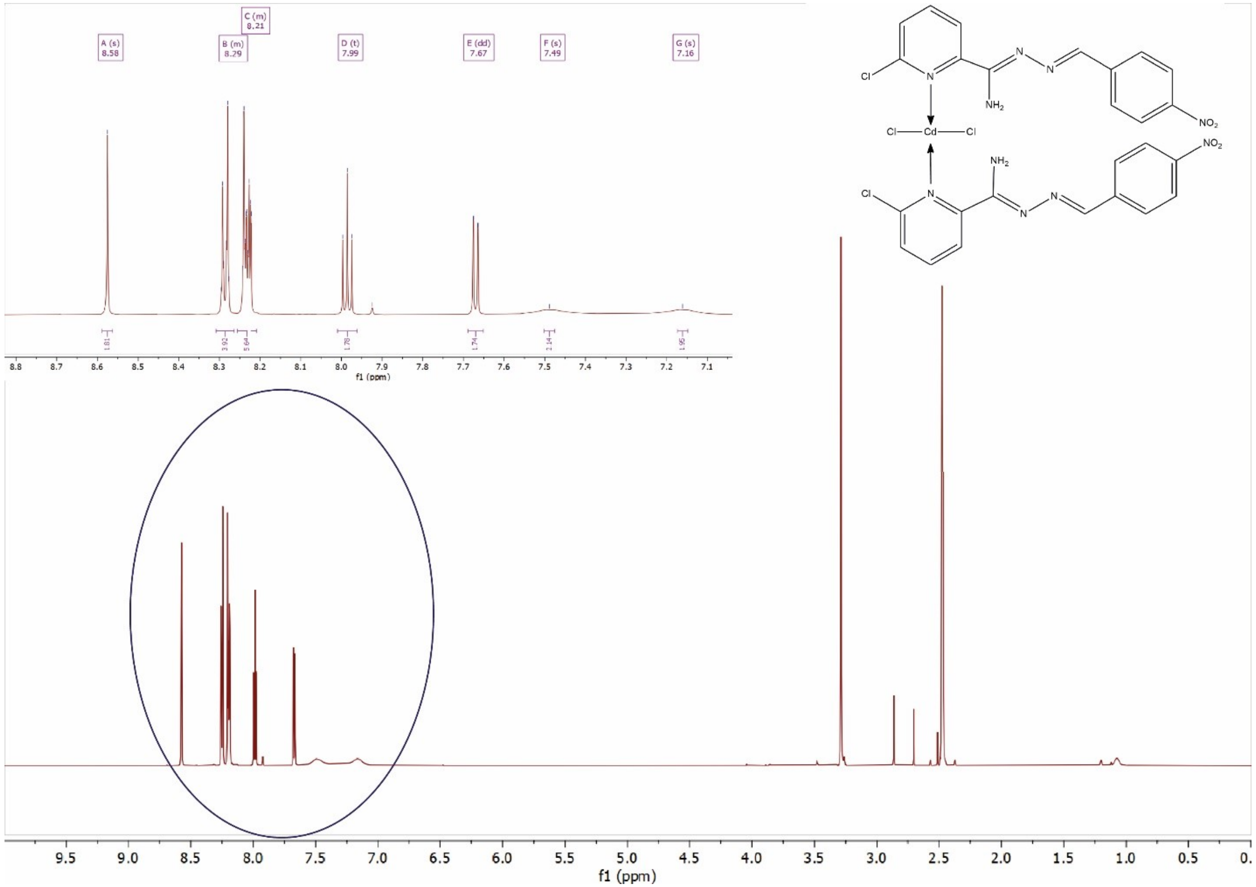

Figure S3 – ^{1}H NMR of Complex **7** (700 MHz, DMSO-d6): d 7.16, 7.49 (2s, 2H, NH2), 7.67 (d, 1H, pyridine), 7.99 (t, 1H, pyridine), 8.21-8.29 (m, 4H Ph + 1H pyridine), 8.58 (s, 1H, CH) ppm.

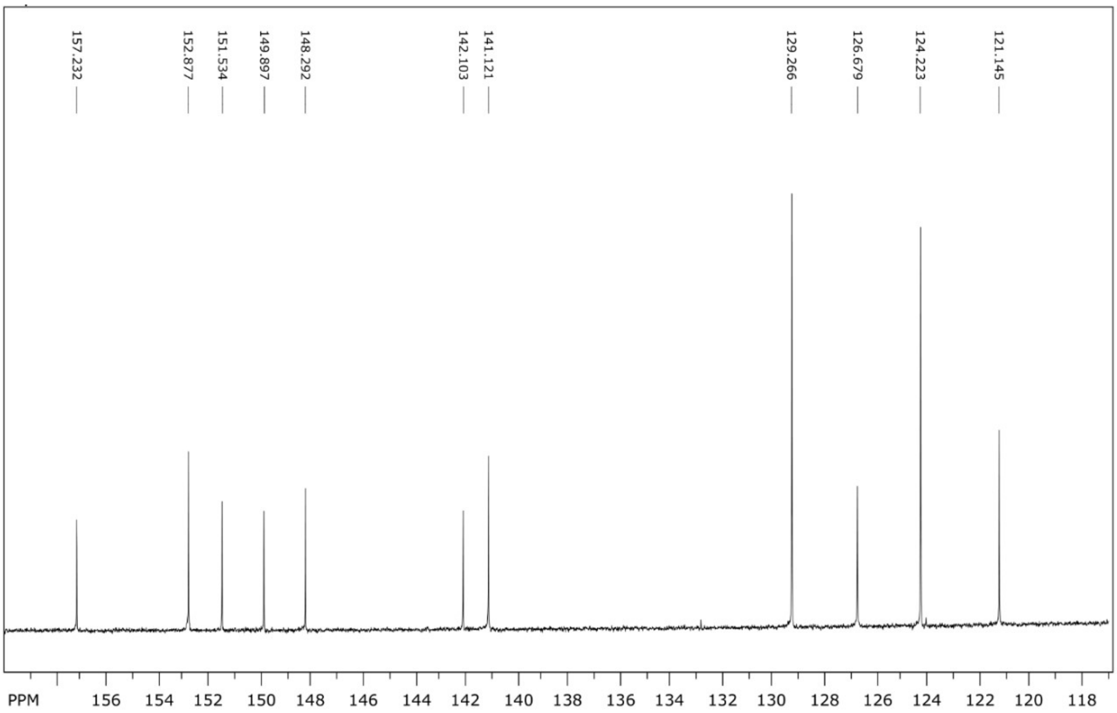

Figure S4 – ^{13}C NMR of L_2 (175 MHz, DMSO-d6): d 121.145, 124.223 (2C), 126.679, 129.266 (2C), 141.121, 142.103, 148.292, 149.897, 151.534, 152.877, 157.232 ppm.

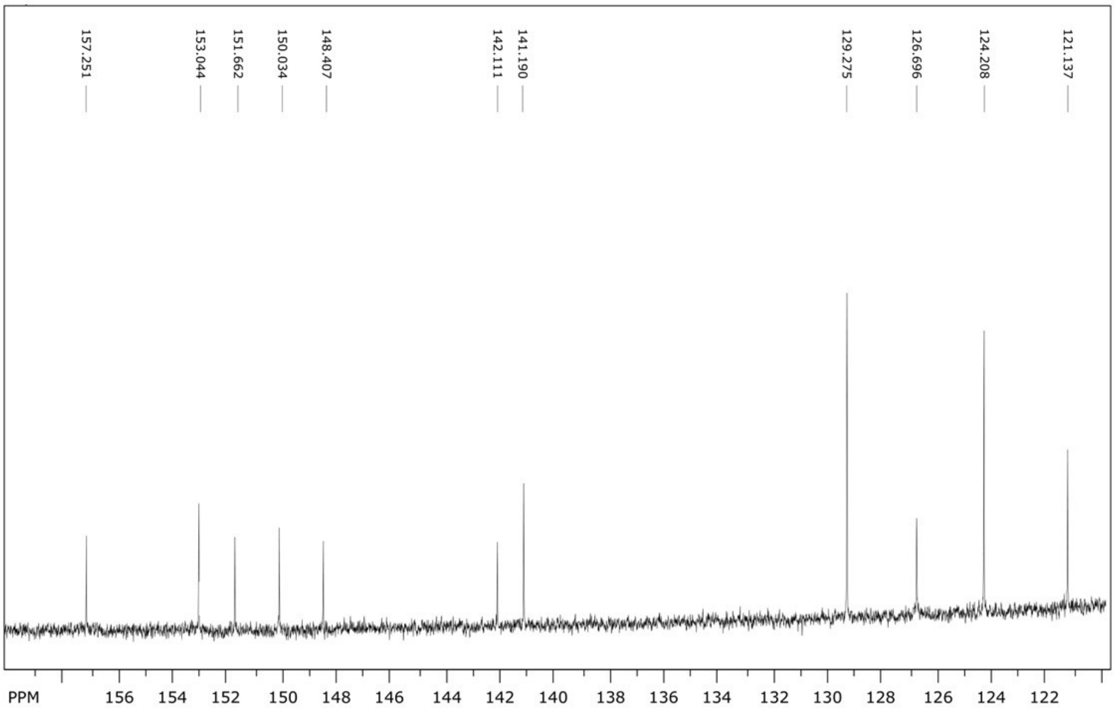

Figure S5 – ^{13}C NMR of Complex **7** (175 MHz, DMSO-d6): d 121.137, 124.208 (2C), 126.696, 129.275 (2C), 141.190, 142.111, 148.407, 150.034, 151.662, 153.044, 157.251 ppm.

Table S2. Experimental details.

Crystal data	
Chemical formula	$C_{34}H_{36}AgN_{13}O_7$
M_r	846.63
Crystal system, space group	Monoclinic, $P2_1/c$
Temperature (K)	100
a, b, c (Å)	11.6968 (2), 16.4562 (2), 18.2059 (3)
β (°)	92.669 (1)
V (Å^3)	3500.56 (9)
Z	4
μ (mm^{-1})	0.65
Crystal size (mm)	0.19 × 0.17 × 0.12
No. of measured, independent and observed [$I > 2\sigma(I)$] reflections	100678, 10869, 9069
R_{int}	0.046
$(\sin \theta/\lambda)_{max}$ (Å^{-1})	0.749
$R[F^2 > 2\sigma(F^2)]$, $wR(F^2)$, S	0.029, 0.067, 1.02
No. of reflections	10869
No. of parameters	496
Δ_{max}, Δ_{min} (e Å^{-3})	0.58, -0.49

Table S3. Geometric parameters (Å, º) for Ag compound.

	Distance [Å]
Ag1—N3A	2.2566(12)
Ag1—N3	2.2627(12)
Ag1—N12	2.5011(12)
Ag1—N12A	2.5094(12)
	Angle [°]
N3A—Ag1—N3	159.45(4)
N3A—Ag1—N12	118.05(4)
N3—Ag1—N12	69.57(4)
N3A—Ag1—N12A	70.32(4)
N3—Ag1—N12A	114.59(4)
N12—Ag1—N12A	147.16(4)

Table S4. Hydrogen-bond geometry (Å, º) for **Ag** complex.

D—H···*A*	*D*—H	H···*A*	*D*···*A*	*D*—H···*A*
N5—H5*B*···O3*I*[i]	0.88	2.08	2.8963 (18)	153
N5*A*—H5*AB*···O4*I*	0.88	2.09	2.8349 (18)	141

Symmetry code: (i) *x*+1, -*y*+1/2, *z*-1/2.

Table S5. Crystal data of complex **6**.

Crystal data	
Empirical formula	$C_{16}H_{18}AgN_7O_3$
Formula weight	464.24
Temperature	119.98(13) K
Wavelength	0.71073 Å
Crystal system, space group	Triclinic, P -1
Unit cell dimensions	a = 7.4241(2) Å α = 92.472(3)°<br>b = 9.5025(3) Å β = 92.340(3)°<br>c = 12.4352(4) Å γ = 95.168(3)°
Volume	872.01(5) $Å^3$
Z, Calculated density	2, 1.768 Mg . m^{-3}
Absorption coefficient	1.190 mm^{-1}
F(000)	468
Crystal size	0.060 x 0.040 x 0.020 mm
Theta range for data collection	1.641 to 31.084°
Limiting indices	-9<=h<=10, -12<=k<=13, -14<=l<=16
Reflections collected / unique	9914 / 4320 [R(int) = 0.0428]
Completeness to theta= 25.242	99.7 %
Absorption correction	Semi-empirical from equivalents
Max. and min. transmission	1.00000 and 0.74682
Refinement method	Full-matrix least-squares on F^2
Data / restraints / parameters	4320 / 195 / 253
Goodness-of-fit on F^2	1.101
Final R indices [I>2σ(I)]	R1 = 0.0554, wR2 = 0.1060
R indices (all data)	R1 = 0.0793, wR2 = 0.1146
Largest diff. peak and hole	1.613 and -1.018 e . $Å^{-3}$

Table S6. Bond distances [Å] and angles [°] for Ag atoms. Symmetry codes: a -x+2,-y+1,-z+1 ; b -x+1,-y+1,-z+2.

Ag1—N2	2.127(4)	N2—Ag1—N2a	180.0
Ag1—N2a	2.127(4)	N5—Ag2—N5b	180.0
Ag2—N5	2.161(3)	N5—Ag2—O1_1	92.20(12)
Ag2—N5b	2.161(3)		
Ag2—O1_1	2.809(3)		

Table S7. Hydrogen-bond geometry [Å, º] for A. Symmetry code: b -x+1,-y+1,-z+2.

D—H···A	d(D—H)	d(H···A)	<DHA	d(D···A)
N3—H3A···O3_1b	0.814	2.054	169.27	2.858
C10—H10···O3_1b	0.950	2.485	131.38	3.193
C11—H11···O1_1	0.950	2.659	136.77	3.414

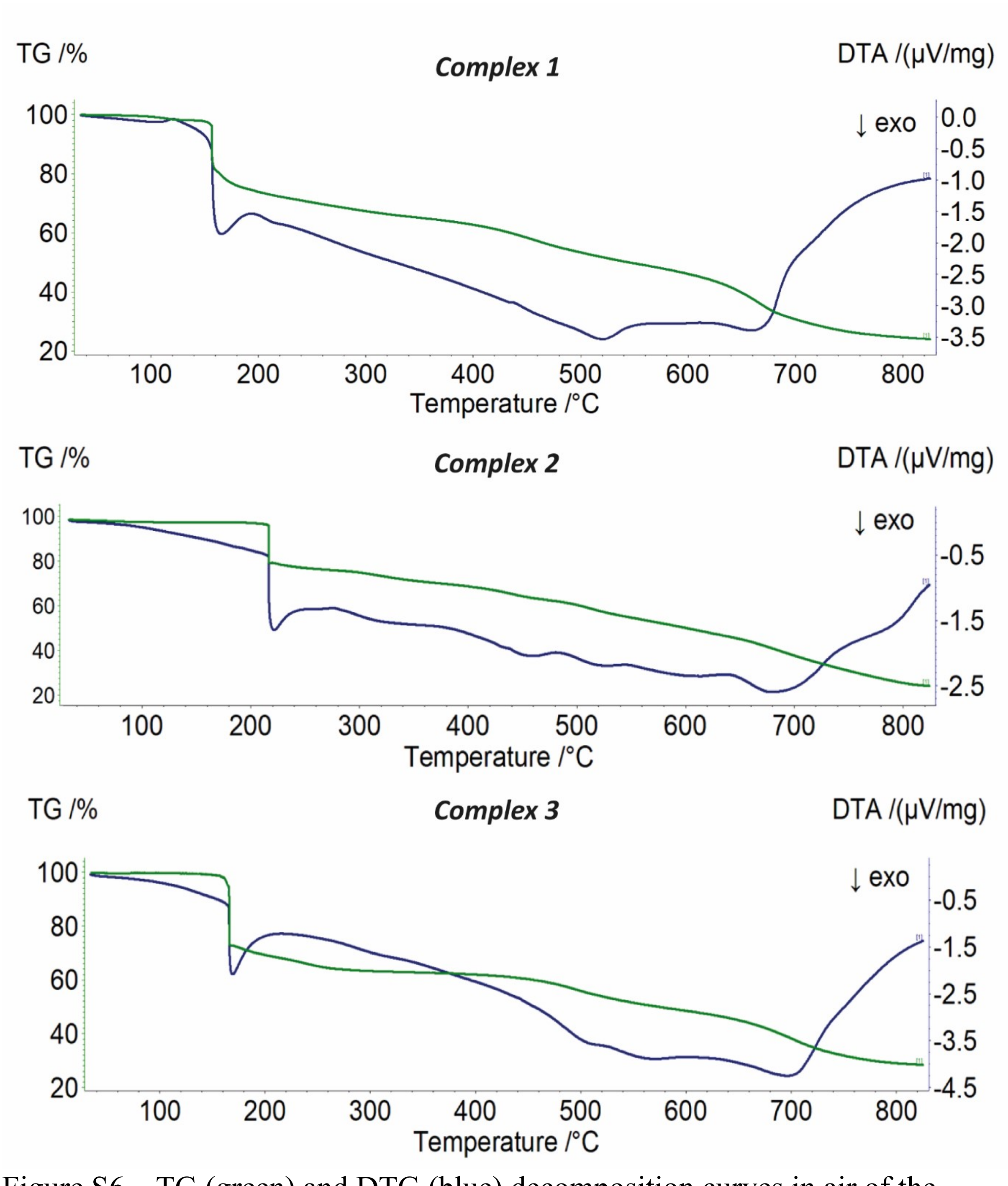

Figure S6 – TG (green) and DTG (blue) decomposition curves in air of the complexes 1, 2, 3.

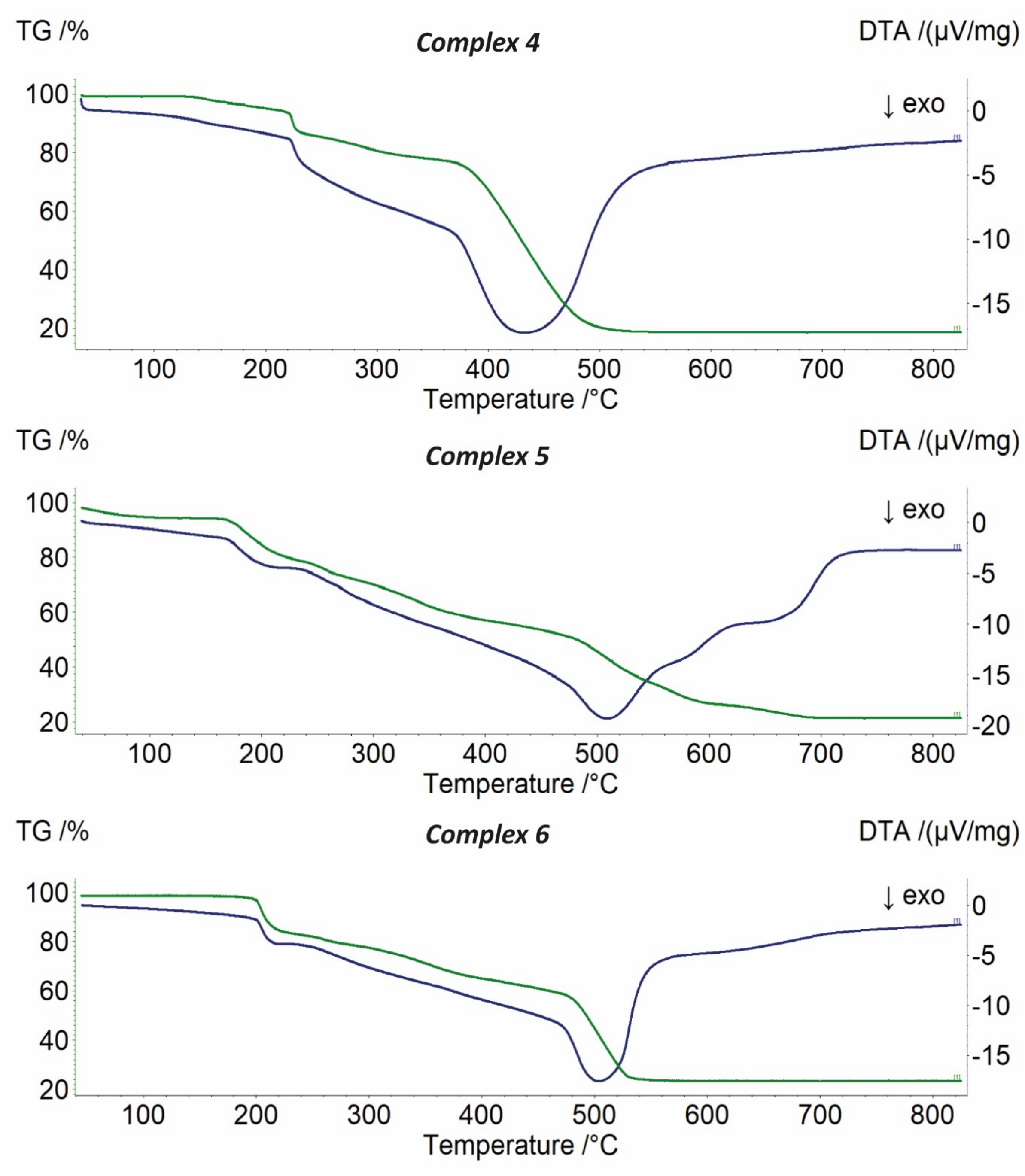

Figure S7 – TG (green) and DTG (blue) decomposition curves in air of the complexes 4, 5, 6.

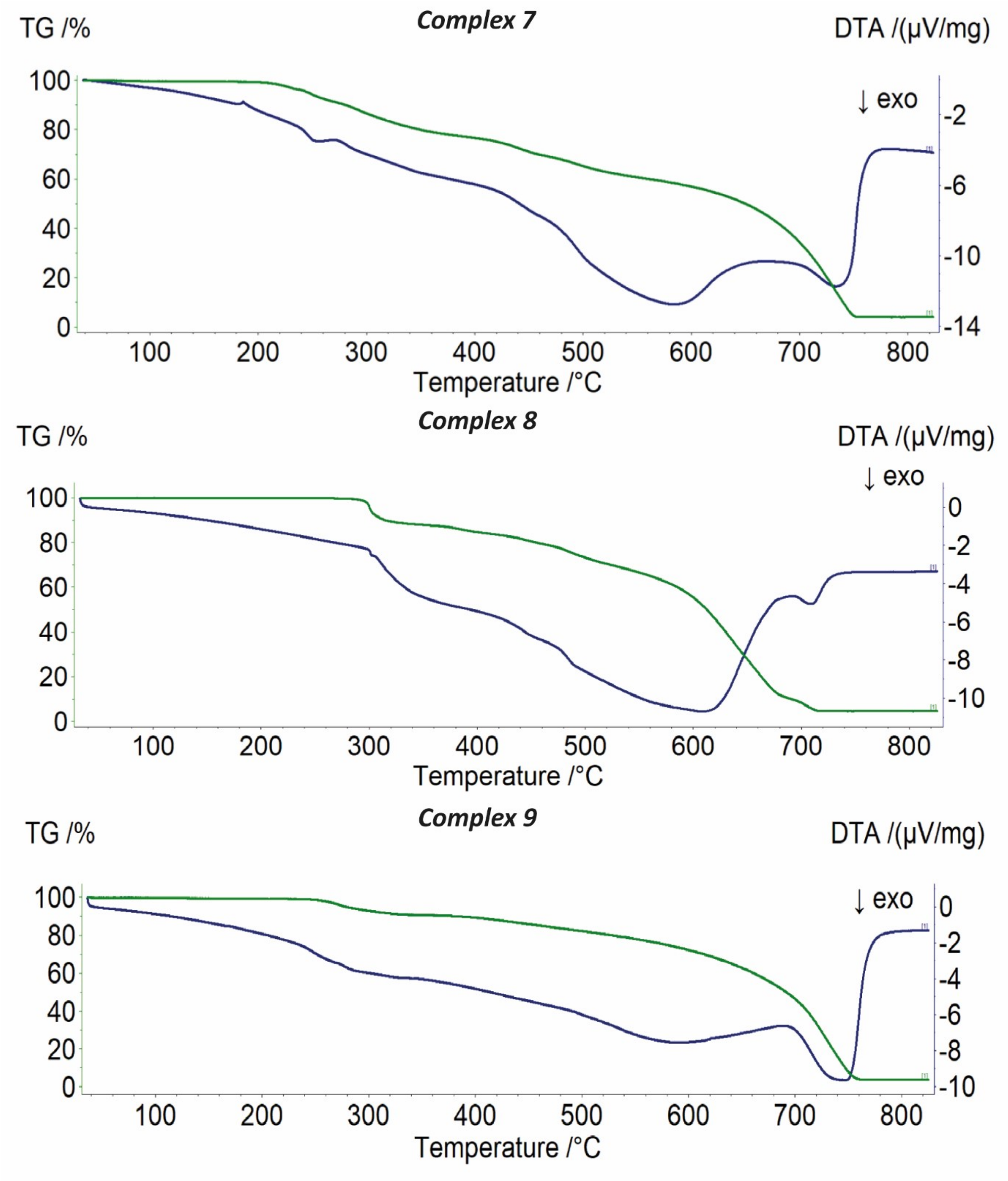

Figure S8 – TG (green) and DTG (blue) decomposition curves in air of the complexes 7, 8, 9.

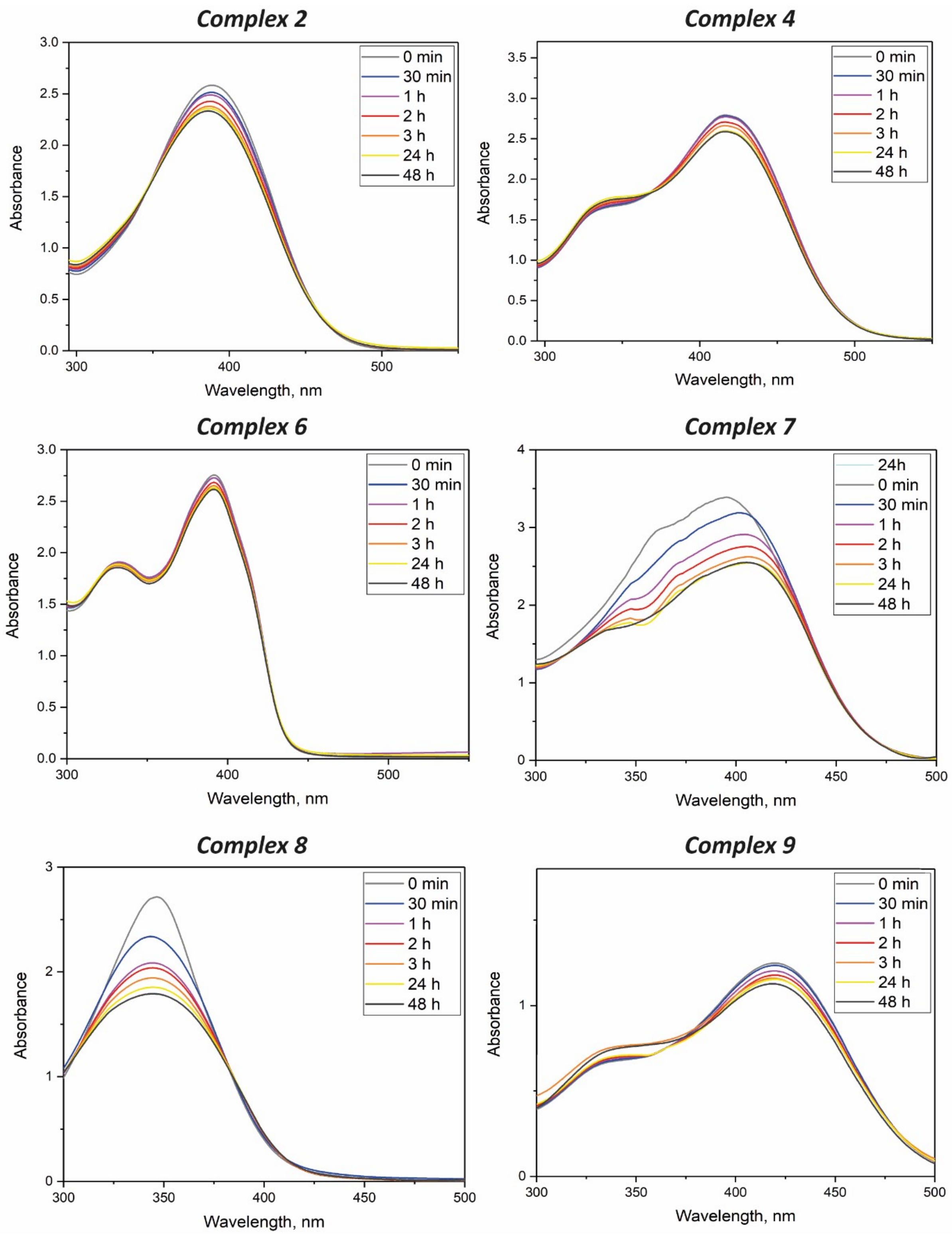

Figure S9 – Time-dependant stability study of the complexes in DMSO.

Table S8. Antimicrobial activity of tested complexes and reference drugs.

**Microorganism**	**Complexes**									**Reference drug**
	1	2	3	4	5	6	7	8	9	
**Gram-positive bacteria**	**MIC [µg/mL]**									**Vancomycin**
Staphylococcus aureus ATCC 25923	<10	50	100	<10	50	50	100	100	10	1
Staphylococcus aureus ATCC 6538	<10	50	50	100	10	10	500	500	250	1
Staphylococcus epidermidis ATCC 12228	10	>500	250	50	<10	<10	>500	>500	>500	1
Enterococcus faecalis ATCC 29212	50	50	50	50	50	>500	>500	>500	10	1
Bacillus cereus ŁOCK 0807	50	50	50	50	50	50	>500	>500	250	1
Listeria monocytogenes ATCC 19115	<10	10	10	10	10	10	>500	>500	500	2
Bacillus subtilis ATCC 6633	50	50	10	50	50	50	>500	>500	>500	1
**Gram-negative bacteria**	**MIC [µg/mL]**									**Ciprofloxacin**
Escherichia coli ATCC 10530	<10	10	10	<10	>500	>500	500	10	10	1
Salmonella Typhimurium ATCC 14028	<10	50	500	100	50	50	>500	>500	>500	1

Table S9. Inhibition zones of the tested complexes in the concentration 500 µg/mL.

**Microorganism**	**Complexes**								
	1	2	3	4	5	6	7	8	9
**Concentration, µg/mL**	500	500	500	500	500	500	500	500	500
**Gram-positive bacteria**	**Inhibition zone, mm**								
S. aureus ATCC 25923	20	19	15	21	19	19	15	15	16
S. aureus ATCC 6538	16	22	15	16	17	18	13	11	13
S. epidermidis ATCC 12228	18	-	11	16	18	17	-	-	-
E. faecalis ATCC 29212	17	14	13	15	17	-	-	-	14
B. cereus ŁOCK 0807	17	18	18	17	19	18	-	-	13
L. monocytogenes ATCC 19115	19	19	19	19	19	18	-	-	12
B. subtilis ATCC 6633	17	16	17	18	18	16	-	-	-
**Gram-negative bacteria**	**Inhibition zone, mm**								
E. coli ATCC 10530	20	20	20	20	-	11	12	20	20
S. Typhimurium ATCC 14028	17	19	11	17	17	17	-	-	-

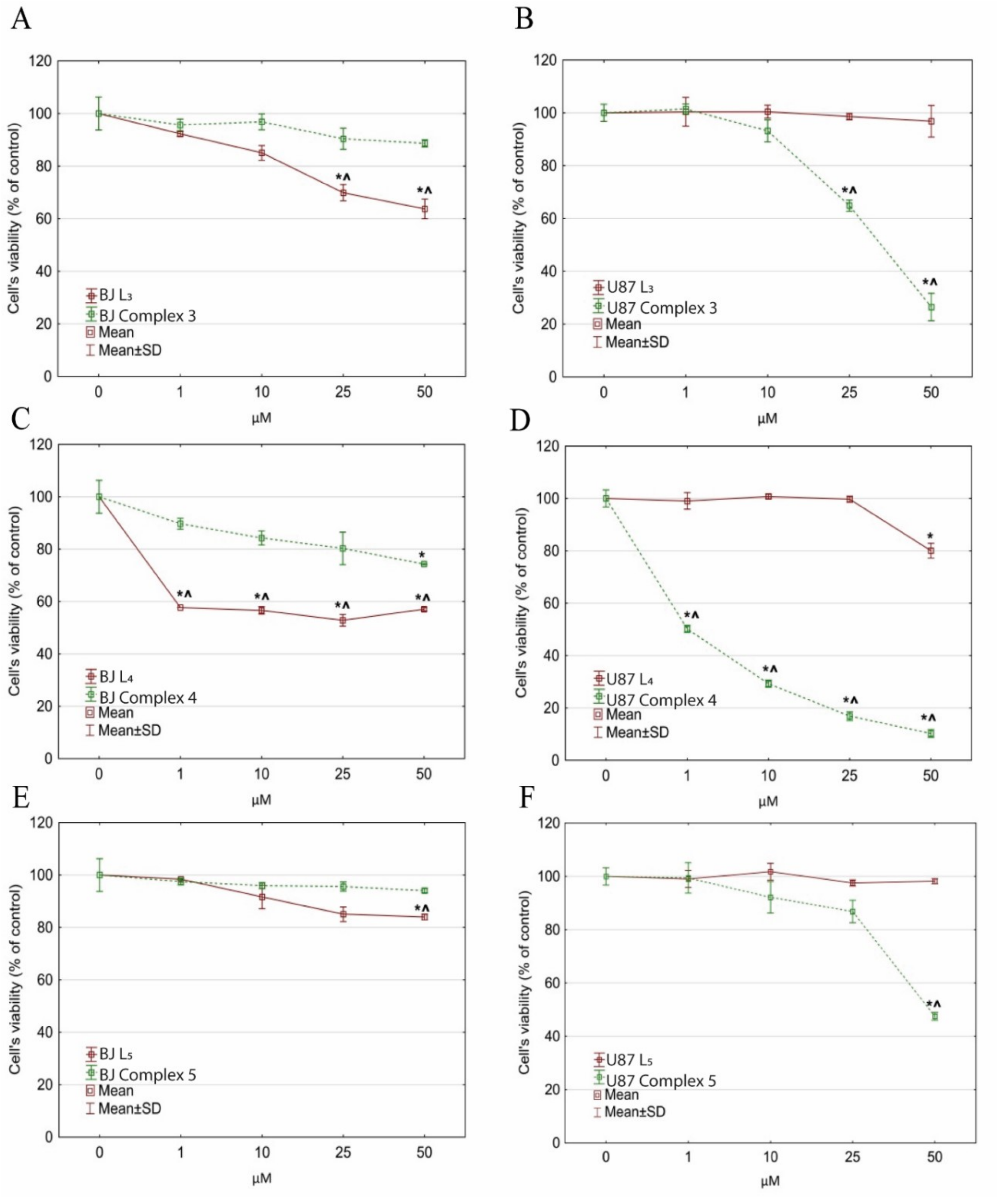

Figure S10 – Cell viability based on MTT test results. A, C, E – viability of normal fibroblast BJ treated with L_3, L_4, L_5 and their Ag complexes **3**, **4**, **5**; B, D, F – viability of glioblastoma U87 cells treated with L_3, L_4, L_5 and their Ag complexes 3, 4, 5. Control cultures (assumed to be 100%) were treated with DMSO as vehicle.

Synthesis of the ligands L_1-L_3

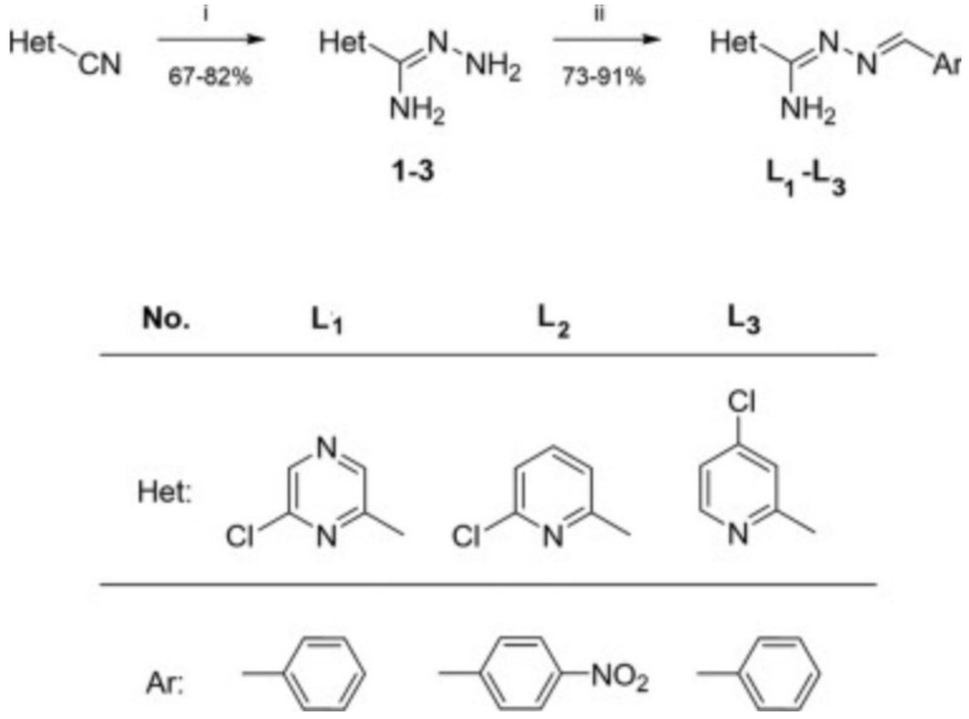

General procedure for the synthesis of hydrazonamides 1–3

The appropriate Het-CN (10 mmol) was dissolved in methanol (20 mL), 99% hydrazine hydrate (5 mL, 103 mmol) was added and the solution was refluxed for 1 h. The solvent was evaporated and placed on the ice bath. The precipitate was filtered off, dried and recrystallized from methanol.

6-chloropyrazine-2-carbohydrazonamide ($C_5H_6ClN_5$) (171.59 g/mol) (1). Starting from 6-chloropyrazine-2-carbonitrile (10 mL), the final compound 1 was obtained as brown solid (11.90 g, 69%): mp 153–156 °C; FTIR spectra (KBr, cm^{-1}): ν(NH) 3412, 3327, 3266; ν(CH) 3158; ν(C=N) 1651; δ(Nsingle bondH) 1577, 1559; ν(C=C) 1445; β(CH) 1178, 1135; γ(CH) 878, 865; ν(CCl) 742. ^{1}H NMR (500 MHz, DMSO-d6): δ 5.84 (s, 2H, NH_2), 5.74 (s, 2H, NH_2), 3.35 (s, 1H, pyrazine), 2.51 (s, 1H, pyrazine) ppm.

6-chloropicolinohydrazonamide ($C_5H_7ClN_4$) (170.60 g/mol) (2). Starting from 6-chloropicolinonitrile (1.39 g), the resulting compound 2 was obtained as white solid (1.40 g, 82%): mp 193–195 °C; FTIR spectra (KBr, cm^{-1}): ν(Nsingle bondH) 3448, 3282; ν(CH) 3171; ν(C=N) 1644; δ(NH) 1583, 1556; ν(C=C) 1455; β(CH) 1164, 1140; γ(CH) 844, 802; ν(CCl) 733. ^{1}H NMR (500 MHz, DMSO-d6): δ 5.51 (s, 2H, NH2), 5.64 (s, 2H, NH2), 7.42 (d, 1H, pyridine, J = 7.5 Hz), 7.79 (t, 1H, pyridine, J = 8 Hz), 7.86 (d, 1H, pyridine, J = 8 Hz) ppm.

4-chloropicolinohydrazonamide ($C_5H_7ClN_4$) (170.60 g/mol) (3). Starting from 4-chloropicolinonitrile (1.39 g), the resulting compound 3 was obtained as white solid (1.15 g, 67%): mp 107–109 °C; FTIR spectra (KBr, cm^{-1}): ν(NH) 3452, 3398, 3348; ν(CH) 3191; ν(C=N) 1610; δ(NH) 1576, 1548; ν(C=C) 1464; β(CH) 1134; γ(CH) 830, 813; ν(CCl) 747. ^{1}H NMR (500 MHz, DMSO-d6): δ 5.49 (s, 2H, NH_2), 5.77 (s, 2H, NH_2), 7.45 (s, 1H, pyridine), 7.90 (s, 1H, pyridine), 8.48 (d, 1H, pyridine, J = 5.5 Hz) ppm.

General procedure for the synthesis of L1-L3 ligands

The appropriate hydrazonamide (1 mmol) was dissolved in methanol (10 mL) and treated with the appropriate aldehyde (1 mmol). The mixture was refluxed for 0.5 h. After cooling, the precipitate was filtered off, dried and recrystallized from a suitable solvent.

N′-(benzylidene)-6-chloropyrazine-2-carbohydrazonamide ($C_{12}H_{10}ClN_5$) (259.69 g/mol) (L_1). Starting from 6-chloropyrazine-2-carbohydrazonamide (0.172 g) and benzaldehyde (0.106 mL), the final ligand L_1 was obtained as yellow solid (0.223 g, 86%): mp 121–123 °C (ethanol); FTIR spectra (KBr, cm^{-1}): ν(NH) 3461, 3332; ν(CH) 3060; ν(CN) 1629; δ(NH) 1556; ν(C=C) 1450; β(CH) 1363; ν(NN) 1166; γ(CH) 881; ν(CCl) 696. ^1H NMR (500 MHz, DMSO-d6): δ 7.22 (s, 2H, NH_2), 7.45–7.46 (m, 3H, Ph), 7.96–7.98 (m, 2H, Ph), 8.53 (s, 1H, CH), 8.93 (s, 1H, pyrazine), 9.33 (s, 1H, pyrazine) ppm; ^{13}C NMR (175 MHz, DMSO-d6): δ 128.61 (2C), 129.06 (2C), 130.74, 135.54, 141.66, 145.79, 146.55, 147.60, 154.87, 156.08 ppm.

6-chloro-N′-(4-nitrobenzylidene)picolinohydrazonamide ($C_{13}H_{10}ClN_5O_2$) (303.70 g/mol) (L_2). Starting from 6-chloropicolinohydrazonamide (0.171 g) and 4-nitrobenzaldehyde (0.227 g), the resulting ligand L_2 was obtained as yellow solid (0.277 g, 91%): mp 192–194 °C (dioxane); FTIR spectra (KBr, cm^{-1}): ν(NH) 3481, 3373; ν(CH) 3078; ν(CN) 1625; δ(NH) 1556; ν(C=C) 1456; ν(NO_2) 1515, 1340; β(CH) 1380; ν(NN) 1016; γ(CH) 875; ν(CCl) 744. ^1H NMR (500 MHz, DMSO-d6): δ 7.22, 7.57 (2 s, 2H, NH_2), 7.71 (d, 1H, pyridine, J = 8 Hz), 8.02 (t, 1H, pyridine, J = 8 Hz), 8.21–8.29 (m, 4H Ph + 1H pyridine), 8.60 (s, 1H, CH) ppm; ^{13}C NMR (175 MHz, DMSO-d6): δ 121.13, 124.20 (2C), 126.68, 129.24 (2C), 141.10, 142.10, 148.26, 149.90, 151.54, 152.86, 157.23 ppm.

N′-(benzylidene)-4-chloropicolinohydrazonamide ($C_{13}H_{11}ClN_4$) (258.71 g/mol) (L_3). Starting from 4-chloropicolinohydrazonamide (0.171 g) and benzaldehyde (0.106 mL), the final ligand L_3 was obtained as yellow solid (0.189 g, 73%): mp 121–122 °C (methanol-water 1:1). FTIR spectra (KBr, cm^{-1}): ν(NH) 3473, 3292; ν(CH) 3056; ν(CN) 1616; δ(NH) 1548; ν(C=C) 1467; β(CH) 1361; ν(NN) 1174; γ(CH) 871; ν(CCl) 692. $_1H$ NMR (500 MHz, DMSO-d6): δ 7.20 (s, 2H, NH2), 7.44–46 (m, 3H, Ph), 7.69–7.71 (m, 1H, pyridine), 7.93–7.95 (m, 2H, Ph), 8.24 (d, 1H, pyridine, J = 2 Hz), 8.52 (s, 1H, CH), 8.66 (d, 1H, pyridine, J = 5.5 Hz) ppm; $_{13}C$ NMR (175 MHz, DMSO-d6): δ 121.40, 125.81, 128.45 (2C), 129.06 (2C), 130.54, 135.71, 144.00, 150.57, 152.75, 155.37, 155.98 ppm.

Synthesis of the ligands L_4-L_6

General procedure for the synthesis of nitriles 1–3.

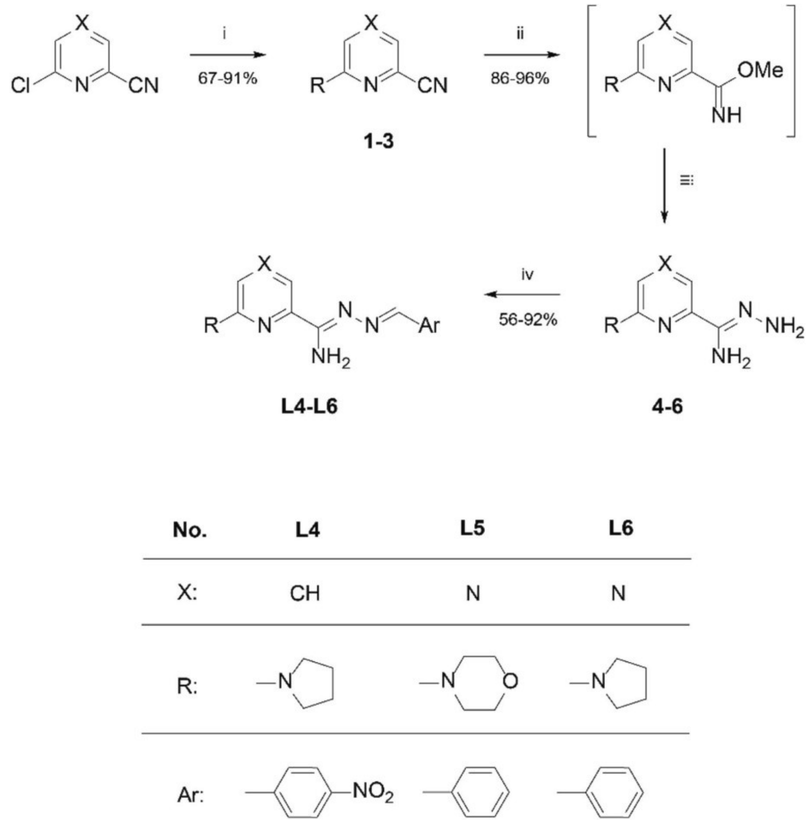

Method A (1). First, 40 mmol of 6-chloropicolinonitrile and 48 mmol of an appropriate nucleophilic agent (pyrrolidine) were dissolved in 25 mL of dioxane, and then, 6 mL of DBU was added. The mixture was refluxed for 1 h. After the evaporation of the solvent , ice was added. The precipitate was filtered and recrystallized using methanol.

Method B (2). First, 40 mmol of 6-chloropyrazine-2-carbonitrile and 48 mmol of an appropriate nucleophilic agent (morpholine) were dissolved in 25 mL of dioxane, and then, 6 mL of DBU was added. The mixture was stirred for 1 h. After the evaporation of the solvent , ice was added. The precipitate was filtered and purified by column chromatography (AcOEt:$CHCl_3$ 2:1).

Method C (3). First, 40 mmol of 6-chloropyrazine-2-carbonitrile and 48 mmol of an appropriate nucleophilic agent (pyrrolidine) were dissolved in 25 mL of dioxane, and then, 6 mL of DBU was added. The mixture was refluxed for 1 h. After the evaporation of the solvent, ice was added, resulting in the formation of an oily suspension, which was neutralized with concentrated hydrochloric acid and then extracted with chloroform (3 × 20 mL). The combined organic layers were dried over anhydrous $MgSO_4$. The drying agent was filtered off, and the solvent evaporated. After triple washing with anhydrous diethyl ether, a precipitate formed which was then purified by column chromatography (AcOEt:$CHCl_3$ 2:1).

6-(pyrrolidin-1-yl)picolinonitrile ($C_{10}H_{11}N_3$) (173.21 g/mol) (1). Starting from 6-chloropicolinonitrile (5.5 g) and morpholine (4 mL), compound 1 was obtained as white crystals (6.3 g, 91%): m.p. 73–75 °C (methanol); IR (KBr): 3097, 3075 (υ C_{Ar}-H), 2959, 2865 (υ C-H), 2230 (υ C≡N), 1616, 1595 (υ C=N), 1499, 1458 (υ C=C), 1247, 1225, 1207 (δ C-H), 793 (γ C-H) cm−1; $_{1}$H NMR (500 MHz, DMSO-d6): δ 1.92–1.95 (m, 4H, 2CH2), 3,37 (t, 4H,

2CH2, J = 7 Hz), 6.75 (d, 1H, pyridine, J = 9 Hz), 7.08 (d, 1H, pyridine, J = 7 Hz), 7.62 (dd, 1H, pyridine, J1 = 8 Hz, J2 = 8 Hz) ppm.

6-morpholinopyrazine-2-carbonitrile ($C_9H_{10}N_4O$) (190.21 g/mol) (2). Starting from 6-chloropyrazine-2-carbonitrile (3.9 mL) and pyrrolidine (4.18 mL), compound 2 was obtained as yellow crystals (6.46 g, 85%): m.p. 116–118 °C (AcOEt:$CHCl_3$ 2:1); IR (KBr): 3067 (υ C_{Ar}-H), 2982, 2912, 2871 (υ C-H), 2237 (υ C≡N), 1575 (υ C=N), 1516, 1446 (υ C=C), 1267, 1231 (δ C-H), 1118, 1068 (υ C O), 873 (γ C-H) cm−1; ^{1}H NMR (500 MHz, DMSO-d6): δ 3.57 (t, 4H, $2CH_2$, J = 5 Hz), 3.68 (t, 4H, $2CH_2$, J = 5 Hz), 8.31 (s, 1H, pyrazine), 8.62 (s, 1H, pyrazine) ppm.

6-(pyrrolidin-1-yl)pyrazine-2-carbonitrile ($C_9H_{10}N_4$) (174.21 g/mol) (3). Starting from 6-chloropyrazine-2-carbonitrile (3.9 mL) and pyrrolidine (4 mL), compound 3 was obtained as yellow crystals (4.65 g, 67%): m.p. 76–78 °C (AcOEt:$CHCl_3$ 2:1); IR (KBr): 3063 (υ C_{Ar}-H), 2979, 2951, 2863 (υ C-H), 2229 (υ C≡N), 1584 (υ C=N), 1519, 1491, 1459 (υ C=C), 1228, 1178, 1161 (δ C-H), 874, 855 (γ C-H) cm^{-1}; ^{1}H NMR (500 MHz, DMSO-d6): δ 1.93–1.95 (m, 4H, $2CH_2$), 2.48 (t, 4H, $2CH_2$, J = 2 Hz), 8.20 (s, 1H, pyrazine), 8.24 (s, 1H, pyrazine) ppm.

General procedure for the synthesis of hydrazonamides 4–6

Method A (4). To a solution of nitrile (10 mmol) in methanol (75 mL), DBU (13.5 mmol, 2 mL) was added, and the mixture was refluxed for 4 h. After the partially evaporation of the solvent, 80% hydrazine hydrate (31.7 mmol, 1 mL) was added and heated at reflux for 15 min. Then, the mixture was poured onto 15 g of ice, and resulting precipitate recrystallized from methanol.

Method B (5,6). To a solution of nitrile (10 mmol) in methanol (75 mL), DBU (13.5 mmol, 2 mL) was added, and the mixture was refluxed for 4 h. After the partially evaporation of the solvent, 80% hydrazine hydrate (31.7 mmol, 1 mL) was added and heated at reflux for 2.5–3.5 h. Then, the mixture was cooled, and resulting precipitate recrystallized from methanol.

6-(pyrrolidin-1-yl)picolinohydrazonamide ($C_{10}H_{15}N_5$) (205.27 g/mol) (4). Starting from 6-(pyrrolidin-1-yl)picolinonitrile (2.05 g), compound 4 was obtained as yellow crystals (1.77 g, 86%): m.p. 223–226 °C (methanol); IR (KBr): 3442, 3377 (υ N-H), 3075 (υ C_{Ar}-H), 2961, 2863 (υ C-H), 1652 (υ C=N), 1595 (δ N-H), 1497, 1458 (υ C=C), 1247, 1159 (δ C-H), 793 (γ C-H) cm^{-1}; ^{1}H NMR (500 MHz, DMSO-d6): δ 1.90–1.93 (m, 4H, 2CH2), 3.34–3.37 (m, 4H, $2CH_2$), 5.11 (br. s, 2H, NH_2), 5.59 (br. s, 2H, NH2), 6.30 (d, 1H, pyridine, J = 8 Hz), 6.74 (d, 1H, pyridine, J = 9 Hz), 7.40 (t, 1H, pyridine, J= 8 Hz) ppm.

6-morpholinopyrazine-2-carbohydrazonamide ($C_9H_{14}N_6O$) (222.25 g/mol) (5). Starting from 6-morpholinopyrazine-2-carbonitrile (2.22 g), compound 5 was obtained as yellow crystals (2.13 g, 96%): m.p. 146–149 °C (methanol); IR (KBr): 3415, 3320 (υ N-H), 3089 (υ C_{Ar}-H), 2963, 2853 (υ C-H), 1648 (υ C=N), 1567 (δ N-H), 1524, 1438 (υ C=C), 1247, 1159 (δ C-H), 1118 (υ C-O), 881 (γ C-H) cm^{-1}; ^{1}H NMR (500 MHz, DMSO-d6): δ 3.55 (t, 4H, $2CH_2$, J = 5 Hz), 3.70 (t, 4H, $2CH_2$, J = 5 Hz), 5.37 (br. s, 2H, NH_2), 5.64 (br. s, 2H, NH_2), 8.11 (s, 1H, pyrazine), 8.34 (s, 1H, pyrazine) ppm.

6-(pyrrolidin-1-yl)pyrazine-2-carbohydrazonamide ($C_9H_{14}N_6$) (206.25 g/mol) (6). Starting from 6-(pyrrolidin-1-yl)pyrazine-2-carbonitrile (2.06 g), compound 6 was obtained as yellow crystals (1.78 g, 86%): m.p. 140–142 °C (methanol); IR (KBr): 3438, 3353(υ N-H), 3075 (υ

CAr-H), 2970, 2864 (υ C-H), 1640 (υ C=N), 1577 (δ N-H), 1519, 1483 (υ C=C), 1208, 1161 (δ C-H), 838 (γ C-H) cm^{-1}; ^{1}H NMR (500 MHz, DMSO-d6): δ 1.92–1.95 (m, 4H, $2CH_2$), 3.44 (t, 4H, 2CH2, J = 7 Hz), 5.31 (br. s, 2H, NH_2), 5.59 (br. s, 2H, NH_2), 7.76 (s, 1H, pyrazine), 8.22 (s, 1H, pyrazine) ppm.

General procedure for the synthesis of ligands L4–L6

The appropriate hydrazonamide (1 mmol) was dissolved in methanol (10 mL) and treated with the appropriate aldehyde (1 mmol). The mixture was refluxed for 0.5–2 h. After cooling, the precipitate was filtered off, dried and recrystallized from a suitable solvent.

L_4 – (4-nitrobenzylidene)-6-(pyrrolidin-1-yl)picolinohydrazonamide ($C_{18}H_{18}N_6O_2$) (338.37 g/mol). Starting from 6-(pyrrolidin-1-yl)picolinohydrazonamide (0.205 g) and 4-nitrobenzaldehyde (0.227 g), compound L_4 was obtained as orange crystals (0.188 g, 56%): m.p. 171–175 °C (ethanol); IR (KBr): 3468, 3339 (υ NH), 3074 (υ C_{Ar}-H), 2972, 2948, 2851 (υ CH), 1619 (υ CN), 1577 (δ NH), 1503, 1474 (υ C=C), 1331 (υ NO_2), 1247, 1153 (δ C H), 802 (γ C H) cm^{-1}; ^{1}H NMR (500 MHz, DMSO-d6): δ 1.97 (t, 4H, $2CH_2$, J = 6 Hz), 3.45–3.49 (m, 4H, $2CH_2$), 6.58 (d, 1H, pyridine, J = 9 Hz), 7.06 (br. s, 1H, NH), 7.37 (br. s, 1H, NH), 7.44 (d, 1H, pyridine, J = 7 Hz), 7.59 (t, 1H, pyridine, J = 8 Hz), 8.18 (d, 2H, Ph, J = 9 Hz), 8.28 (d, 2H, Ph, J = 9 Hz), 8.56 (s, 1H, CH) ppm; ^{13}C NMR (125 MHz, DMSO-d6): δ 25.45 (2C), 46.78 (2C), 108.93, 109.06, 124.19 (2C), 128.87 (2C), 137.98, 142.46, 148.00, 148.34, 151.48, 156.41, 159.01 ppm.

L_5 – (benzylidene)-6-morpholinopyrazine-2-carbohydrazonamide ($C_{16}H_{18}N_6O$) (310.36 g/mol). Starting from 6-morpholinopyrazine-2-carbohydrazonamide (0.222 g) and benzaldehyde (0.106 mL), compound L_5 was obtained as yellow crystals (0.237 g, 77%): m.p. 205–207 °C (ethanol); IR (KBr): 3453, 3304 (υ NH), 3053, 3022 (υ C_{Ar}-H), 2972, 2842 (υ CH), 1613 (υ CN), 1564 (δ NH), 1526, 1444 (υ C=C), 1262, 1215 (δ CH), 1122 (υ CO), 864 (γ CH) cm^{-1}; ^{1}H NMR (500 MHz, DMSO-d6): δ 3.66 (t, 4H, $2CH_2$, J = 5 Hz), 3.74 (t, 4H, 2CH2, J = 5 Hz), 7.08 (br. s, 2H, NH_2), 7.44–7.45 (m, 3H, Ph), 7.93 (dd, 2H, Ph, J1 = 2 Hz, J2 = 2 Hz), 8.39 (s, 1H, pyrazine), 8.49 (s, 1H, pyrazine), 8.63 (s, 1H, CH) ppm; ^{13}C NMR (125 MHz, DMSO-d6): δ 44.72 (2C), 66.29 (2C), 128.35 (2C), 129.00 (2C), 137.98, 130.38, 130.81, 132.33, 135.82, 142.93, 153.77, 154.79, 156.47 ppm.

L_6 – (benzylidene)-6-(pyrrolidin-1-yl)pyrazine-2-carbohydrazonamide ($C_{16}H_{18}N_6$) (294.36 g/mol). Starting from 6-(pyrrolidin-1-yl)picolinohydrazonamide (0.206 g) and benzaldehyde (0.106 mL), compound L_6 was obtained as yellow crystals (0.271 g, 92%): m.p. 211–213 °C (ethanol); IR (KBr): 3450, 3317 (υ NH), 3055, 3020 (υ C_{Ar}-H), 2864 (υ CH), 1614 (υ CN), 1564 (δ NH), 1526, 1437 (υ C=C), 1233, 1159 (δ CH), 860 (γ CH) cm^{-1}; ^{1}H NMR (500 MHz, DMSO-d6): δ 1.98 (t, 4H, $2CH_2$, J = 2 Hz), 3.53 (t, 4H, 2CH2, J = 7 Hz), 6.84 (br. s, 1H, NH), 7.11 (br. s, 1H, NH_2), 7.43–7.45 (m, 3H, Ph), 7.92 (dd, 2H, Ph, J1 = 2 Hz, J2 = 2 Hz), 8.04 (s, 1H, pyrazine), 8.48 (s, 1H, pyrazine), 8.51 (s, 1H, CH) ppm; ^{13}C NMR (125 MHz, DMSO-d6): δ 25.31 (2C), 46.55 (2C), 128.29 (2C), 128.83, 129.00 (2C), 130.34, 132.12, 135.84, 143.10, 151.76, 154.65, 156.60 ppm.
